# Mu Opioid Receptor Positive Allosteric Modulator BMS-986122 Confers Agonist-Dependent G Protein Subtype Signaling Bias

**DOI:** 10.1021/acs.biochem.5c00022

**Published:** 2025-05-16

**Authors:** Grant M. Grieble, Brian I. Knapp, Jean M. Bidlack

**Affiliations:** Department of Pharmacology & Physiology, 12299 University of Rochester School of Medicine and Dentistry, Rochester, New York 14642, United States

## Abstract

The mu opioid receptor (MOR) is a G protein-coupled receptor (GPCR) and is responsible for the effects of all medically used opioids. Most opioids activate all inhibitory Gαi/o/z proteins through MOR, initiating signaling events that culminate in a variety of physiological effects such as analgesia, euphoria, and respiratory depression. Gaining a better understanding of how the chemical structure of opioids influences the functional activation profiles of G protein subtypes by MOR is critical for disentangling the multitude of opioid effects and the development of safer analgesics. A recent development in opioid pharmacology has been the discovery of positive allosteric modulators (PAMs) for opioid receptors, such as BMS-986122, which act at the MOR to increase the potency of full agonists and the efficacy of partial agonists. Here, we utilized a nanoBRET-based functional assay system in live HEK 293T cells to study how the pharmacological properties of opioids were uniquely affected by BMS-986122 when the MOR signaled through specific inhibitory Gα subunits. We report that BMS-986122 differentially enhanced opioid activity when the MOR signaled through different Gα subunits with the greatest difference observed with partial agonists. Additionally, the binding affinity of BMS-986122 to the MOR was significantly altered by the co-binding Gα subunit. Site-directed mutagenesis experiments revealed key amino acid residue differences on Gαi/o subunits involved in the differential effects observed. This study sheds light on the molecular features of biased signaling for both opioid ligands and G proteins, which may prove useful for the further development of biased agonists or allosteric modulators at the MOR.

## Graphical abstract

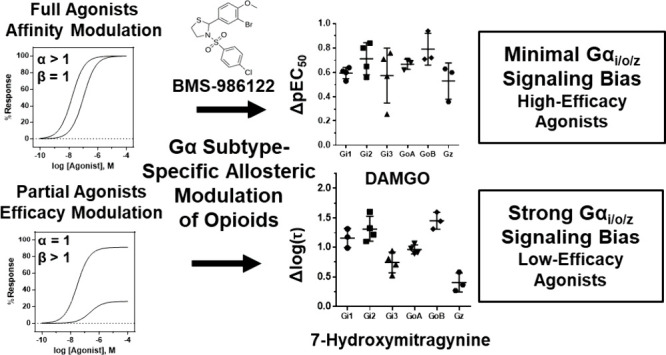

## Introduction

The mu opioid receptor (MOR) is a class A G protein-coupled receptor (GPCR) that is responsible for eliciting the multitude of effects of opioid drugs, including analgesia, euphoria, and respiratory depression by initiating intracellular signaling cascades through the activation of heterotrimeric G proteins.^1–3^ In heterologous expression systems, the MOR is able to activate all inhibitory G proteins, Gαi1, Gαi2, Gαi3, GαoA, GαoB, and Gαz, which results in the modulation of enzyme systems such as adenylyl cyclase, kinases, and ion channels such as G protein-activated inwardly rectifying K^+^ channels.^4–7^ At present, the precise mechanisms of how these signaling events culminate into the wide variety of opioid effects are not clear. Interestingly, there is growing evidence that the six inhibitory Gα proteins, while highly homologous, participate in nonoverlapping signaling networks through differential effector engagement and display unique tissue distributions across the CNS and periphery.^8–12^ However, whether the activation of specific G protein subtypes results in the therapeutic effects of opioids or if there is redundancy in their function has yet to be fully established. A promising approach to investigate this has been through the quantitative study of biased agonism, the phenomenon by which certain agonists select GPCR receptor conformational states that allow for the preferential recruitment of some transducer proteins over others.^13,14^ Research into this field has shown that orthosteric ligands for a variety of different GPCRs, including opioid receptors, impart the receptor with unique coupling preferences for specific transducer proteins, including different G protein subtypes, and this may confer physiological effects that are agonist-dependent.^4,8,15,16^ The study of biased signaling applied to inhibitory G protein subtypes has broad implications for GPCRs that activate Gαi/o/z proteins outside of opioid receptors as well including adenosine, dopamine, serotonin, and other receptor subtypes. Knockout studies have provided evidence that indicate inhibitory G protein subtypes display an overall lack of redundancy in their physiological functions *in vivo*.^17–19^ For instance, in the context of opioid pharmacology, the specific genetic deletion of Gαo attenuated opioid-induced antinociception in mice, while deletion of Gαi2 or Gαi3 comparatively had no effect.^17^

The capacity for biased signaling has also been observed for allosteric modulators (AMs) as well, which has been given the term biased allosteric modulation.^20–22^ AMs are ligands that bind to a receptor site that is topographically distinct from the orthosteric binding site and either positively or negatively modify the affinity and/or efficacy of the orthosteric ligand.^23^ Allosteric modulation of the MOR has emerged as an area of substantial interest due to the ability of AMs to “fine tune” the amplitude of receptor signaling without loss of the natural signaling tone of endogenous opioid peptides, as their binding is typically noncompetitive with orthosteric ligands.^24,25^ This unique feature may contribute to the often improved safety profile of GPCR allosteric modulators in humans.^26,27^ Indeed, the MOR positive allosteric modulator (PAM) BMS-986122 enhanced the analgesic effects of the endogenous opioid peptide met-enkephalin with a reduced risk of morphine-like side effects, including constipation and respiratory depression, in mice.^28^ BMS-986122 as well as other allosteric ligands, including BMS-986187 and BMS-986124 were discovered through high-throughput screening campaigns using the PathHunter enzyme complementation assay.^24,29^ While the pharmacological properties of BMS-986122 have been generally characterized at the MOR,^24,30,31^ its precise effects on opioid signaling when MOR signals through specific Gα protein subtypes was not extensively studied.

Here, we employed a nanoBRET-based functional assay system^32^ in live HEK 293T cells to study the pharmacological effects of BMS-986122 on various MOR full and partial agonists. The benefit of BRET-based assays, compared to other functional assays such as the [^35^S]­GTPγS assay, comes from the fact that a single Gα protein subtype may be transiently overexpressed to study the pharmacological properties of opioids when MOR signals through that specific G protein. We report that the specific Gα subunit coupling to the MOR significantly altered the degree to which BMS-986122 modulated either the potency or the efficacy of MOR agonists, and this effect was highly agonist-dependent. Additionally, the calculated affinity constant of BMS-986122 (K_B_) was shown to vary substantially depending on which Gα subunit was coupled to the MOR. For each agonist tested, the Gα subunit bias imparted by BMS-986122 on opioid signaling was quantified using both the Black–Leff operational model of agonism^33,34^ as well as the functional allosteric model,^23,35^ allowing for both a rigorous scale of G protein subtype bias to be established as well as an estimation of system-independent allosteric parameters such as affinity cooperativity (α), efficacy cooperativity (β), and PAM affinity (K_B_), for BMS-986122. Calculations of G protein-subtype bias of BMS-986122 were additionally shown to be consistent across both the nanoBRET assay and TruPATH,^36^ an orthogonal BRET-based assay system. Lastly, the characterization of Gα protein C-terminal α5 helix mutants revealed specific amino acid residues involved in the G protein subtype bias conferred by BMS-986122. Altogether, this study contributes to the growing knowledge of structure–activity relationships in GPCR-biased signaling and indicates that MOR allosteric modulators may be further designed and optimized to confer the specific activation of G protein subtypes, potentially allowing the signaling repertoire of the MOR to be restricted to therapeutically relevant effects *in vivo*.

## Materials and Methods

### Opioid Alkaloids and Peptides

[d-Ala 2, N-MePhe 4, Gly-ol]-enkephalin (DAMGO) acetate and methionine-enkephalin (met-enkephalin) acetate were obtained from Bachem (Bubendorf, Switzerland). (−)-Pentazocine^37,38^ was obtained from Dr. Mark Wentland (Rensselaer Polytechnic Institute, Troy, NY). 7-Hydroxymitragynine^39^ was obtained from Cayman Chemical (Ann Arbor, MI). Morphine sulfate was obtained from the Mallinckrodt Chemical Company (St. Louis, MO). BMS-986122 ^24^ was obtained from MedChem Express (Monmouth Junction, NJ). BMS-986187 ^29^ was obtained from Tocris Bioscience (Bristol, UK). BMS-986124 ^24,31^ was obtained from Sigma-Aldrich Corp. (St. Louis, MO). [N-allyl-2,3-^3^H]­Naloxone (79.9 Ci/mmol) was obtained from Revvity, Inc. (Waltham, MA).

### Membrane Preparations for Radioligand Binding Assays

Chinese hamster ovary cells stably expressing the human MOR (MOR-CHO; obtained from Dr. George Uhl, NIDA Intramural Research Program, Baltimore, MD) were cultured in 10 cm dishes in Dulbecco’s modified Eagle’s medium (DMEM) supplemented with 5% fetal bovine serum and 100 U/mL penicillin–streptomycin at 37 °C in a 10% CO_2_ atmosphere. Cell membranes were isolated and homogenized as previously described,^40^ and membrane protein concentrations were determined with bovine serum albumin as the control as previously described.^41^ Membranes were stored at −80 °C until use.

### Radioligand Competition Binding Assays

To determine the effects of BMS-986122 on the affinity of MOR full and partial agonists, 12 different concentrations of each compound were incubated for 60 min with MOR-CHO cell membranes (50 μg membrane protein) in a low-affinity buffer^40^ (50 mM Tris-HCl, pH 7.4, 100 mM NaCl, 5 mM MgCl_2_, 1 mM EDTA, 1 mM DTT, and 50 μM GDP) in the absence and presence of 10 μM BMS-986122. [^3^H]­Naloxone was used at a final concentration of 1.0 nM, and nonspecific binding was measured using 10 μM unlabeled naloxone. Membranes were added last to the incubation tube. All assays were performed at 25 °C and terminated by rapid vacuum filtration through Whatman #32 glass fiber filters using a Brandel cell harvester (Biomedical Research and Development Laboratories Inc., Gaithersburg, MD) followed by three ice-cold washes with 50 mM Tris-HCl, pH 7.4. Samples were collected and counted in 2 mL of Ecoscint A scintillation fluid (National Diagnostics, Atlanta, GA) for 2 min each using a LS 6500 scintillation counter (Beckman Coulter Inc., Fullerton, CA).

### Cell Culture, Plasmids, and Transfection

Human embryonic kidney (HEK) 293T cells (ATCC, Manassas, VA) maintained and transfected as described in previous work,^42^ with some modifications. Cells were cultured on 0.01% poly-l-lysine (Millipore Sigma, Darmstadt, Germany)-coated 10 cm dishes in Dulbecco’s modified Eagle medium (GIBCO, Grand Island, NY) supplemented with 5% fetal bovine serum, 1% nonessential amino acids, 1 mM sodium pyruvate, and 100 U/mL penicillin/streptomycin, maintained in an incubator at 37 °C in a 5% CO_2_ atmosphere. Prior to transfection, 3.0 × 10^6^ cells were reseeded onto Matrigel-coated (Corning, Inc., Corning NY) 6 cm dishes and incubated for 4 h at 37 °C and 5% CO_2_. The human MOR (cDNA Resource Center, Bloomsburg, PA), human Gα subunit of interest (cDNA Resource Center, Bloomsburg, PA), mVenus­(156-239)-Gβ_1_, mVenus­(1-155)-Gγ_2_, and myristic acid attachment sequence (mas) with the C-terminus of G protein-coupled receptor kinase 3 (GRK3ct) fused with nanoluciferase (masGRK3ct-nLuc;^32^ gifts from Dr. Kirill A. Martemyanov, The Scripps Research Institute Florida, Jupiter, FL) were transfected at a 1:2:1:1:1 ratio (ratio 1 = 0.42 μg plasmid DNA) as previously described.^32,42^ Lipofectamine LTX with PLUS reagent (Invitrogen, Carlsbad, CA) in antibiotic-free OptiMEM I Reduced Serum Medium (GIBCO, Grand Island, NY) was utilized for transfections. When RGS20 (cDNA Resource Center, Bloomsburg, PA) was cotransfected with the other five constructs, 0.42 μg of plasmid DNA was used.

For TruPATH assay experiments, 3.0 × 10^6^ cells plated on 6 cm dishes were transfected with the human MOR, human Gα subunit of interest fused with rLuc8, Gβ_3_, and Gγ_8_ or Gγ_9_ fused with GFP2 (purchased from Dr. Bryan Roth’s TRUPATH pcDNA5/FRT/TO Addgene library^36^), at a 1:1:1:1 ratio (ratio 1 = 0.63 μg plasmid DNA) using Lipofectamine LTX with PLUS reagent.

For both nanoBRET and TruPATH assays, after 18–24 h, transfected cells were reseeded onto 0.01% poly-l-lysine (Millipore Sigma)-coated solid white 96-well flat bottom plates (Greiner Bio-One North America, Inc., Monroe, NC) at approximately 50,000–75,000 cells/well with a final volume of 150 μL of DMEM supplemented with 10% fetal bovine serum, 1% nonessential amino acids, 1 mM sodium pyruvate, and 100 U/mL penicillin/streptomycin. For both nanoBRET and TruPATH, cells were incubated for an additional 18–24 h at 37 °C and 5% CO_2_ on the 96-well plate, which was observed to give a higher ΔBRET ratio compared to the procedure employed in Barnett et al.^42^ in which BRET measurements are made the day after transfection, thus improving the dynamic range of the assay system.

### Measuring MOR Signaling Through Different Gα Subunits

BRET measurements between mVenus-Gβ_1_γ_2_ and masGRK3ct-nLuc for nanoBRET, and Gγ-GFP2 and Gα-rLuc8 for TruPATH, were performed to determine agonist-dependent activation of each Gα protein of interest in the presence and absence of BMS-986122, in live HEK 293T cells. After the second 18–24 h incubation at 37 °C and 5% CO_2_, media was shaken off the 96-well plate and cells were washed once with 50 μL PBS (GIBCO) to remove residual media. Twenty-five μL of BRET buffer (PBS with 0.5 mM MgCl_2_ and 0.1% glucose) with or without BMS-986122 at varying concentrations was added to each well, followed by 25 μL Nano-Glo Luciferase Assay Substrate (Promega, Madison, WI) diluted 1:50 in BRET buffer for nanoBRET, or 25 μL Prolume Purple (NanoLight Technologies, Pinetop, AZ) diluted 1:50 in a BRET buffer for TruPATH. Lastly, 50 μL of opioid was added at varying concentrations, and cells were incubated for 5 min at 25 °C prior to luminescence emissions being read on a BMG POLARstar Omega Microplate Reader (BMG Labtech, Ortenberg, Germany) at 535 and 475 nm for nanoBRET. For TruPATH, cells were incubated for 30 min at 25 °C prior to luminescence emissions being read at 515 and 410 nm. Each assay was performed in duplicate and repeated with separate transfections at least three times. A baseline with no opioid stimulation was set as the minimum BRET signal, and 10 μM DAMGO, a MOR full agonist, was set as the maximum BRET signal. The mean baseline BRET ratio was subtracted from each experimental BRET ratio to obtain an ΔBRET ratio. All ΔBRET ratios were normalized to the 10 μM DAMGO ΔBRET ratio, which was set at 100%.

### Logistic Curve Fitting and Statistical Analysis

For competition binding experiments, the specific binding of the radioligand in the absence of other competing compounds was set at 100%. The percent of control binding was calculated for increasing concentrations of the competing compounds. A three- or four-parameter logistic curve was fit to the data by using SigmaPlot 11 (Systat Software Inc., San Jose, CA). The IC_50_ values, the concentration of the opioid needed to inhibit 50% of the control binding, were calculated from the curves. K_i_ values were calculated as described previously.^43^ Statistical differences between K_i_ values were calculated using a two-tailed Student’s *t* test. Receptor binding data were reported as the mean K_i_ value ± SEM from three separate experiments performed in triplicate.

For BRET experiments, concentration–response curves were generated in SigmaPlot 11, and E_max_ and EC_50_ values were calculated from a three- or four-parameter logistic curve fit. Data were expressed as the mean EC_50_ and E_max_ values ± SEM from three or more independent experiments performed in duplicate. All figure graphs were constructed using GraphPad Prism 10 (GraphPad Software Inc., San Diego, CA).

### Allosteric and Operational Model Fitting and Statistical Analysis

To derive relative transduction coefficients (Δlog­(τ)) between MOR partial agonists in the presence of vehicle and 10 μM BMS-986122 for each Gα subunit, concentration–response data were fit to the Black–Leff operational model of agonism^33^ using the ‘Operational Model – receptor depletion’ function embedded in GraphPad Prism 10,

1 $$\mathrm{Response}=\mathrm{Basal}+\frac{E_{m}-\mathrm{Basal}}{1+{\left(\frac{{(10}^{\log K_{A}}+10^{\left[A\right]})}{10^{\log\left(\tau \right)+\left[A\right]}}\right)}^{n}}$$

where E_m_ = maximum system response (set to 100%), Basal = response in absence of agonist, n = transducer slope, K_A_ = affinity of agonist, τ = operational efficacy of the agonist, and [A] = concentration of agonist. This form of the operational model was selected because all parameters are constrained by default to be equal for each data set except for τ, which had no constraints. Since BMS-986122 did not affect the binding affinity (K_A_) of partial agonists and only efficacy, these parameter constraints were appropriate for the calculation of relative transduction coefficients. The relative transduction coefficient Δlog­(τ) is defined as,

2 $$\Delta \log\left(\tau \right)={\log\left(\frac{\tau }{K_{A}}\right)}_{10\mathrm{\mu M}\mathrm{BMS}-986122}-{\log\left(\frac{\tau }{K_{A}}\right)}_{\mathrm{Vehicle}}$$

where τ is the operational efficacy of the orthosteric agonist and K_A_ is the affinity of the agonist. Calculating the relative transduction coefficient between vehicle and 10 μM BMS-986122 treatments causes the denominator (K_A_) to cancel out, as they are equal, leaving Δlog­(τ). Statistical significance between Δlog­(τ) values for each Gα subunit were calculated via one-way ANOVA with Tukey’s posthoc analysis. To determine statistical differences between Δlog­(τ) values comparing nanoBRET and TruPATH assay systems, a two-tailed Student’s *t* test was used. To obtain allosteric parameters α (PAM affinity cooperativity factor) and K_B_ (PAM binding affinity) for BMS-986122 for specific Gα subunits when DAMGO and met-enkephalin were the orthosteric agonists, concentration–response data for each agonist in the absence and presence of varying concentrations of BMS-986122 were globally fit to the ‘Allosteric EC_50_ Shift’ function embedded in GraphPad Prism 10, a simplification of the functional allosteric equation for the action of a PAM on a full agonist in which the PAM has no effect on agonist efficacy.^35^ To obtain allosteric parameters β (PAM efficacy cooperativity factor) and K_B_ for BMS-986122 for specific Gα subunits when (−)-pentazocine and 7-hydroxymitragynine were the orthosteric agonists, concentration–response data for each agonist in the absence and presence of varying concentrations of BMS-986122 were globally fit to the functional allosteric equation^23,35^ shown below, using GraphPad Prism 10,

3 $$\frac{E}{E_{m}}=\frac{{(\tau_{A}\left[A\right]\left(K_{B}+\alpha \beta \left[B\right]\right)}^{n}}{{\left(\tau_{A}\left[A\right]\left(K_{B}+\alpha \beta \left[B\right]\right)\right)}^{n}+{\left(\left[A\right]K_{B}+K_{A}K_{B}+K_{A}\left[B\right]+\alpha \left[A\right]\left[B\right]\right)}^{n}}$$

where E_m_ = maximum system response (set to 100%), n = transducer slope, [A] = concentration of agonist, [B] = concentration of modulator, τ_A_ = operational efficacy of agonist, K_A_ = affinity of agonist, K_B_ = affinity of modulator, α = affinity cooperativity factor (set to 1.0 for partial agonists), and β = efficacy cooperativity factor. All model parameter estimates were expressed as the mean value ± SEM from at least three independent experiments performed in duplicate.

### Site-Directed Mutagenesis Experiments

In order to determine specific amino acid residues on Gα subunits involved in producing the differential effects of BMS-986122 on opioid signaling, site-directed mutagenesis was performed on wild-type (WT) Gαi3 on pcDNA 3.1­(+) and WT Gαi2 on pcDNA 3.1­(+) (cDNA Resource Center, Bloomsburg PA) using the Q5 Site-Directed Mutagenesis Kit (New England Biolabs, Ipswich, MA). The kit was followed according to the manufacturer’s instructions. Primer pairs were designed as follows: For the Gαi3 K349R, E350G mutant: fwd: 5′-CAACT­TAAGG­GGATG­TGGAC­TTTAT-3′, rev: 5′-TTTTT­AATGA­TGACA­TCTGT­AACA­GC-3′. For the Gαi3 mutant, in which the C-terminus was replaced with that of GαoB, two additional amino acids on the Gαi3^K349R,E350G^ mutant were altered: K345A and N346 K. The primer pairs were designed as follows: fwd: 5′-GCAA­AAAA­CTTA­AGGG­GATG­TG-3′, rev: 5′-AATGA­TGACA­TCTGT­AACAG­CATC-3′. For the Gαi2 N346 K mutant, the primers were designed as follows: fwd: 5′-AAAAA­CCTGA­AGGA­CTGC-3′, rev: 5′-CTTGA­TGAT­GACA­TCGG-3′.

## Results

### Competition Binding Experiments with MOR Agonists and BMS-986122

We first investigated the effects of 10 μM, a saturating concentration of BMS-986122,^24^ on the binding affinity of opioids for the MOR to determine if BMS-986122 enhanced opioid binding. Utilizing MOR-CHO membrane homogenates, we performed competition binding experiments with [^3^H]­naloxone as the radioligand in a low-affinity buffer (see Materials and Methods) with two full agonists, DAMGO and the endogenous opioid peptide methionine-enkephalin (met-enkephalin), as well as two partial agonists, (−)-pentazocine and 7-hydroxymitragynine. The results of these experiments are shown in Figure 1. Similar to what was reported by Livingston and Traynor,^30^ we observed that 10 μM BMS-986122 induced a leftward shift of the binding curve of both full agonists DAMGO and met-enkephalin (Figures 1A and 1B, respectively), shifting the K_i_ value from 240 ± 31 nM to 35 ± 7.4 nM for DAMGO and from 290 ± 37 nM to 48 ± 13 nM for met-enkephalin. However, BMS-986122 had no effect on the binding affinity of the two partial agonists (−)-pentazocine and 7-hydroxymitragynine (Figures 1C and 1D, respectively). The K_i_ values and their fold-changes obtained from these experiments for all agonists are listed in Table 1.

**Figure 1:**
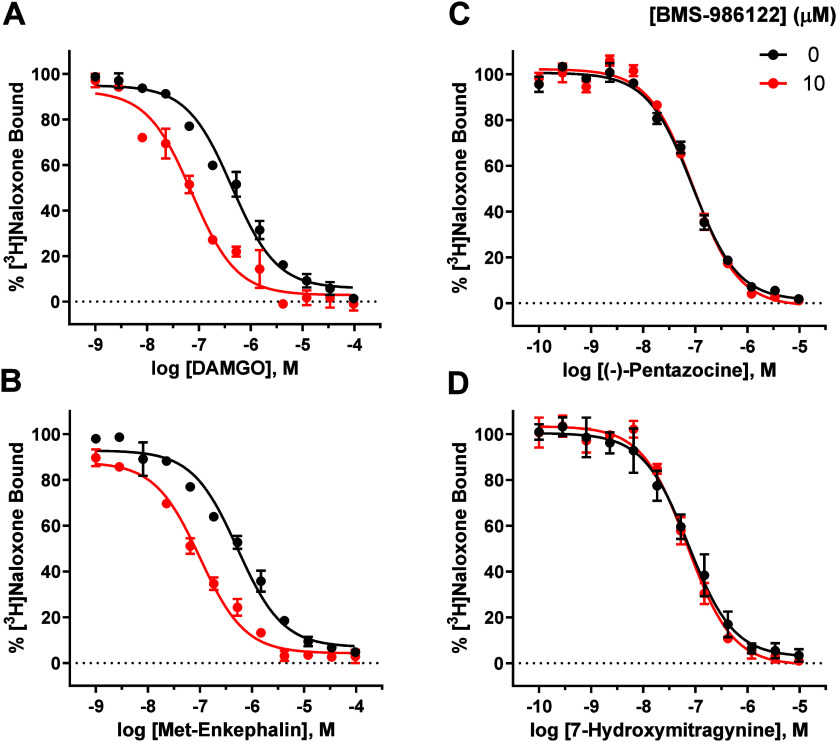
BMS-986122 increased the affinity of full agonists but had no effect on partial agonist binding. [^3^H]­Naloxone binding to CHO cell membranes stably expressing the human MOR (MOR-CHO) was measured in the presence of increasing concentrations of full agonists DAMGO (A) and met-enkephalin (B), and partial agonists (−)-pentazocine (C) and 7-hydroxymitragynine (D), treated either with vehicle (●) or 10 μM BMS-986122 ( red ●). All points are the mean values ± SEM from at least three independent experiments performed in triplicate.

**Table 1:** Inhibition of [^3^H]­Naloxone Binding to MOR by Full and Partial Agonists in the Absence and Presence of 10 μM BMS-986122^a^

Compound	K_i_ (Vehicle) (nM ± SEM)	K_i_ (10 μM BMS) (nM ± SEM)	K_i_ (Veh)/K_i_ (BMS)
DAMGO	240 ± 31	35 ± 7.4	6.8 ****
Met-Enkephalin	290 ± 37	48 ± 13	6.0 **
(−)-Pentazocine	54 ± 5.8	53 ± 4.9	1.0 ns
7-Hydroxymitragynine	43 ± 4.6	40 ± 3.8	1.1 ns

a Affinity (K_i_) values were determined by measuring the displacement of [^3^H]­naloxone in CHO cell membranes stably expressing the MOR, treating with either vehicle or 10 μM BMS-986122, as described in Materials and Methods. Student’s two-tailed *t* test was performed between pK_i_ values obtained when membranes were incubated with vehicle and 10 μM BMS-986122; ** denotes *p* < 0.01, **** *p* < 0.0001, ns *p* > 0.05. Data shown are the mean values ± SEM from at least three independent experiments performed in triplicate.

### Effect of BMS-986122 on G Protein Signaling Properties of MOR Full Agonists

Using a nanoBRET assay system in HEK 293T cells (Figure S1), we investigated the effects of a range of concentrations of BMS-986122 on the pharmacological properties of DAMGO and met-enkephalin when MOR signaled through different Gα subunits. Given the results from our binding experiments described above, we anticipated that BMS-986122 would increase the potency of full agonists without any additional increase in maximal stimulation in a functional assay system, reflecting the capacity of the PAM to increase the binding affinity of full agonists. The ability to transiently overexpress a single Gα protein subtype allowed for the determination of the signaling properties of opioid agonists and BMS-986122 that were unique to specific subtypes of G proteins. Using this system, we were therefore able to determine whether the extent of full agonist affinity modulation by BMS-986122 was altered by the coupling of specific Gα subunits to the MOR.

Concentration–response curves were generated for DAMGO and met-enkephalin in the absence and presence of 10 μM BMS-986122 for each Gα subunit, and average E_max_ and EC_50_ values were calculated, which are listed in Table 2. In the absence of BMS-986122, the potency of both DAMGO and met-enkephalin was similar compared for each Gα subunit MOR signaled through. However, when MOR signaled through Gαz, the potency of both agonists was the greatest. Figure 2 displays representative concentration–response curves of DAMGO in the presence of vehicle, 0.1, 1, and 10 μM BMS-986122 when MOR signaled through three subunits: Gαi1 (Figure 2A), Gαi2 (Figure 2B), and GαoA (Figure 2C). On average, the maximal increase in potency (EC_50_) caused by BMS-986122 was 4.5-fold for DAMGO and 3.8-fold for met-enkephalin. Notably, a comparison of the effects of BMS-986122 on full agonist potency when MOR was signaled through different Gα subunits revealed only minor differences. Potency ratios (ΔpEC_50_ values) between vehicle-treated and 10 μM BMS-986122-treated responses were calculated for each Gα subunit (Table 3). Differences between ΔpEC_50_ values for DAMGO when MOR signaled through different Gα subunits were modest and not sufficient to reach statistical significance (Figure S2A). Turning our attention to met-enkephalin, concentration–response curves in the presence of vehicle, 0.1, 1, and 10 μM BMS-986122 again when MOR signaled through Gαi1 (Figure 2D), Gαi2 (Figure 2E), and GαoA (Figure 2F), were constructed. Differences between potency ratios were narrow for met-enkephalin as well (Table 3); however, the ΔpEC_50_ value when MOR signaled through Gαi1 was significantly greater than Gαi2 and GαoA (*p* < 0.01 and *p* < 0.05, respectively) (Figure S2B). Overall, these results indicate that the cobinding Gα subunit only moderately alters the extent of full agonist affinity modulation of BMS-986122. The extent of the difference appears to be agonist-dependent, however, with DAMGO showing no significant differences among the six Gα subunits.

**Figure 2:**
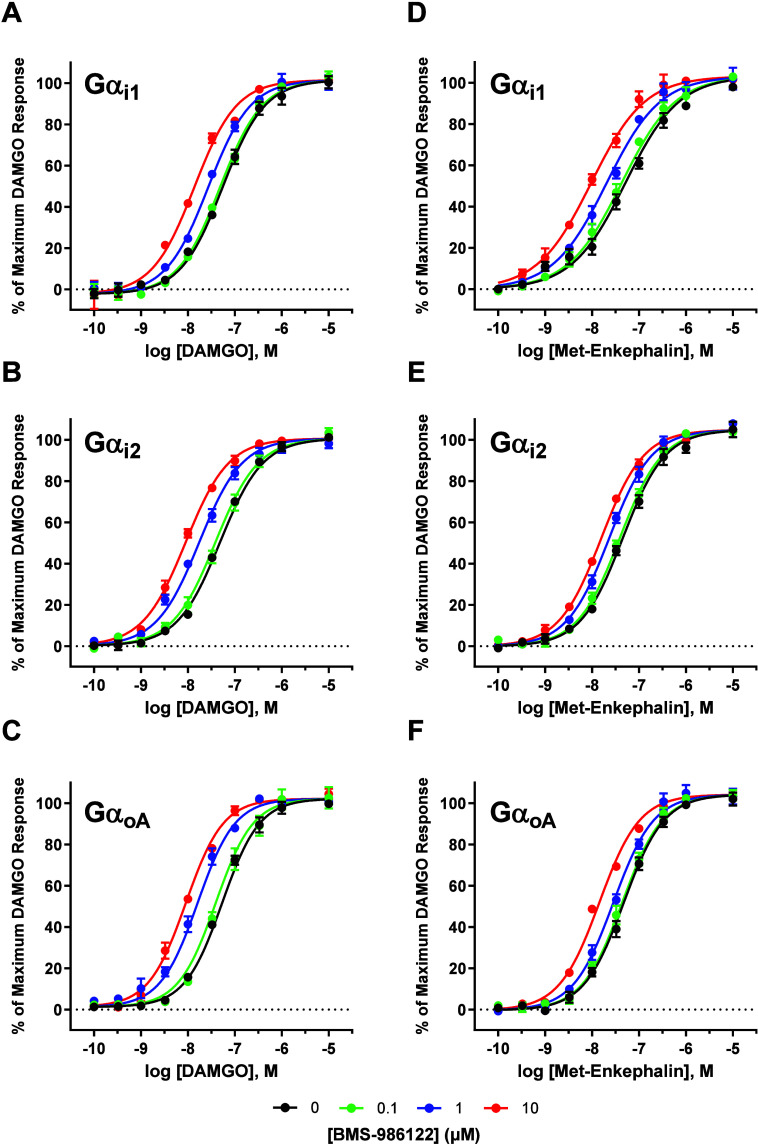
Effect of BMS-986122 on the potency of full agonists when MOR signaled through different Gα subunits. Concentration–response curves were generated for the two full agonists DAMGO and met-enkephalin using the nanoBRET assay in HEK 293T cells transiently expressing the human MOR, Gα subunit of interest, Gβγ-mVenus, and masGRK3_ct_-nLuc, in the presence of vehicle (●), 0.1 μM (green ●), 1.0 μM (blue ●), and 10 μM (red ●) BMS-986122. Increasing concentrations of BMS-986122 induced an increasing leftward shift of the DAMGO concentration–response curve when MOR signaled through Gαi1 (A), Gαi2 (B), and GαoA (C). A similar effect was observed for met-enkephalin when MOR signaled through Gαi1 (D), Gαi2 (E), and GαoA (F). All points are the mean values ± SEM from at least three independent experiments performed in duplicate.

**Table 2:** EC_50_ and E_max_ Values for MOR Full Agonists in the Absence and Presence of 10 μM BMS-986122 when MOR Signaled through Different Gα Subunits^a^

	DAMGO		DAMGO and 10 μM BMS-986122		Met-Enkephalin		Met-Enkephalin and 10 μM BMS-986122	
Subunit	EC_50_ (nM ± SEM)	E_max_ (% ± SEM)	EC_50_ (nM ± SEM)	E_max_ (% ± SEM)	EC_50_ (nM ± SEM)	E_max_ (% ± SEM)	EC_50_ (nM ± SEM)	E_max_ (% ± SEM)
Gαi1	58 ± 4.4	101 ± 3.4	15 ± 1.1	101 ± 1.4	52 ± 4.7	100 ± 2.6	10 ± 1.9	105 ± 3.6
Gαi2	46 ± 4.4	101 ± 1.1	9.1 ± 1.1	100 ± 1.3	45 ± 4.2	104 ± 3.6	15 ± 0.9	103 ± 1.5
Gαi3	24 ± 3.0	104 ± 2.6	7.3 ± 1.2	103 ± 1.7	38 ± 3.5	100 ± 2.6	8.7 ± 0.6	105 ± 2.4
GαoA	45 ± 2.3	100 ± 1.5	10 ± 0.6	105 ± 0.9	53 ± 9.4	104 ± 2.2	15 ± 1.5	103 ± 2.3
GαoB	74 ± 8.0	99 ± 1.5	11 ± 1.3	102 ± 1.2	53 ± 1.3	103 ± 0.9	14 ± 1.6	101 ± 0.3
Gαz	10 ± 0.3	102 ± 3.2	3.2 ± 0.7	108 ± 2.3	35 ± 5.4	103 ± 2.0	17 ± 1.5	104 ± 2.0

a The nanoBRET assay was performed as described in Materials and Methods. Data shown are the mean values ± SEM from at least three independent experiments performed in duplicate.

**Table 3:** ΔpEC_50_ Values for MOR Full Agonists Were Calculated between Treatment with Vehicle and 10 μM BMS-986122 When MOR Signaled through Different Gα Subunits^a^

Subunit	DAMGO ΔpEC_50_ ± SEM	Met-Enkephalin ΔpEC_50_ ± SEM
Gαi1	0.596 ± 0.023	0.723 ± 0.049
Gαi2	0.712 ± 0.064	0.475 ± 0.017
Gαi3	0.467 ± 0.104	0.639 ± 0.034
GαoA	0.667 ± 0.022	0.537 ± 0.038
GαoB	0.791 ± 0.109	0.578 ± 0.056
Gαz	0.529 ± 0.087	0.315 ± 0.091

a Statistical analysis of differential potency modulation of full agonists by BMS-986122 is shown in Figure S2. Data shown are the mean values ± SEM from at least three independent experiments performed in duplicate.

### Effect of BMS-986122 on G Protein Signaling Properties of MOR Partial Agonists

Consequently, we turned our attention to the effects of BMS-986122 on the signaling properties of partial agonists. Concentration–response curves were initially generated for two partial agonists: the benzomorphan (−)-pentazocine and the indole-based alkaloid 7-hydroxymitragynine present in *Mitragyna speciosa* (kratom). The average E_max_ and EC_50_ values were calculated for both partial agonists in the absence and presence of 10 μM BMS-986122 when MOR signaled through all six inhibitory Gα subunits and are listed in Table 4. Unlike DAMGO and met-enkephalin, both E_max_ and EC_50_ values were more varied between the different Gα subunits MOR signaled through for both (−)-pentazocine and 7-hydroxymitragnine alone, and again, potency as well as efficacy were greatest when MOR signaled through Gαz. The addition of 10 μM BMS-986122 increased both the E_max_ value and the EC_50_ of partial agonists at most subunits tested. Because we did not observe any change in affinity of both (−)-pentazocine and 7-hydroxymitragynine by BMS-986122 in the binding experiments, the effect of BMS-986122 on these agonists must result only from an increase in agonist efficacy. In order to quantify the differential effects of BMS-986122 on partial agonist efficacy depending on which Gα subunit MOR signaled through, we fitted our concentration–response data to the Black–Leff operational model of agonism^33,34^ to calculate transduction coefficients (log­(τ/K_A_)) for both (−)-pentazocine and 7-hydroxymitragynine, as well as morphine, in the absence and presence of 10 μM BMS-986122. The difference between the transduction coefficients calculated for each agonist in the presence and absence of 10 μM BMS-986122 (Δlog­(τ/K_A_)) was calculated for each Gα subunit, and these values were directly compared to determine PAM bias factors. Because BMS-986122 did not alter the affinity of partial agonists for the MOR, the K_A_ term (agonist binding affinity) in the denominator canceled out, allowing for relative transduction coefficients to be expressed as Δlog­(τ). Normally, the relative activity (log­(E_max_/EC_50_)) of an agonist may be used for these calculations, as under usual conditions relative activities are approximately equal to transduction coefficients.^13,44^ However, (−)-pentazocine concentration–response curves often displayed Hill slopes that were significantly different than unity, and average E_max_ values of 7-hydroxymitragynine were <30% for most subunits tested, causing relative activity values to diverge from the more stable transduction coefficients.^34,44^ Therefore, we opted to use the operational model to quantify differential PAM effects on the partial agonist efficacy.

**Table 4:** EC_50_ and E_max_ Values for MOR Partial Agonists in the Absence and Presence of 10 μM BMS-986122 When MOR Signaled through Different Gα Subunits^a^

	(−)-Pentazocine		(−)-Pentazocine and 10 μM BMS-986122		7-Hydroxymitragynine		7-Hydroxymitragynine and 10 μM BMS-986122	
Subunit	EC_50_ (nM ± SEM)	E_max_ (% ± SEM)	EC_50_ (nM ± SEM)	E_max_ (% ± SEM)	EC_50_ (nM ± SEM)	E_max_ (% ± SEM)	EC_50_ (nM ± SEM)	E_max_ (% ± SEM)
Gαi1	62 ± 6.4	56 ± 1.4	22 ± 3.2	105 ± 1.2	54 ± 8.8	38 ± 3.9	32 ± 1.9	101 ± 2.0
Gαi2	61 ± 4.1	35 ± 2.0	45 ± 2.5	66 ± 4.3	250 ± 22	20 ± 2.5	72 ± 7.5	77 ± 3.1
Gαi3	20 ± 2.3	70 ± 1.8	12 ± 1.1	102 ± 1.8	130 ± 42	20 ± 2.0	36 ± 10	61 ± 4.3
GαoA	55 ± 3.6	48 ± 2.2	32 ± 4.3	88 ± 3.0	120 ± 27	25 ± 0.9	52 ± 2.7	86 ± 0.9
GαoB	36 ± 2.9	74 ± 0.33	18 ± 4.6	96 ± 2.6	180 ± 27	12 ± 3.3	87 ± 4.1	77 ± 3.2
Gαz	9.7 ± 1.0	105 ± 1.8	5.3 ± 1.2	110 ± 6.7	67 ± 6.5	33 ± 3.0	83 ± 16	65 ± 4.1

a The nanoBRET assay was performed as described in Materials and Methods. Data shown are the mean values ± SEM from at least three independent experiments performed in duplicate.

The relative transduction coefficients between vehicle and 10 μM BMS-986122 treatment for all partial agonists when MOR signaled through different Gα subunits are given in Table 5. Figure 3 shows representative concentration–response curves for (−)-pentazocine treated with vehicle and a range of concentrations of BMS-986122, up to 10 μM, when MOR signaled through Gαi1 (Figure 3A), Gαi2 (Figure 3B), and GαoA (Figure 3C). Comparing the differential effects of 10 μM BMS-986122 on (−)-pentazocine, efficacy modulation was far more pronounced when MOR signaled through Gαi1 (Δlog­(τ) = 0.865 ± 0.048), a significant difference compared to all remaining subunits (Figure S2C), with Gαi2 displaying a much weaker increase in efficacy in comparison (Δlog­(τ) = 0.399 ± 0.043) (*p* < 0.001). BMS-986122 had the weakest effect on efficacy when MOR signaled through Gαz (Δlog­(τ) = 0.239 ± 0.100), a trend that was observed for most agonists tested.

**Figure 3:**
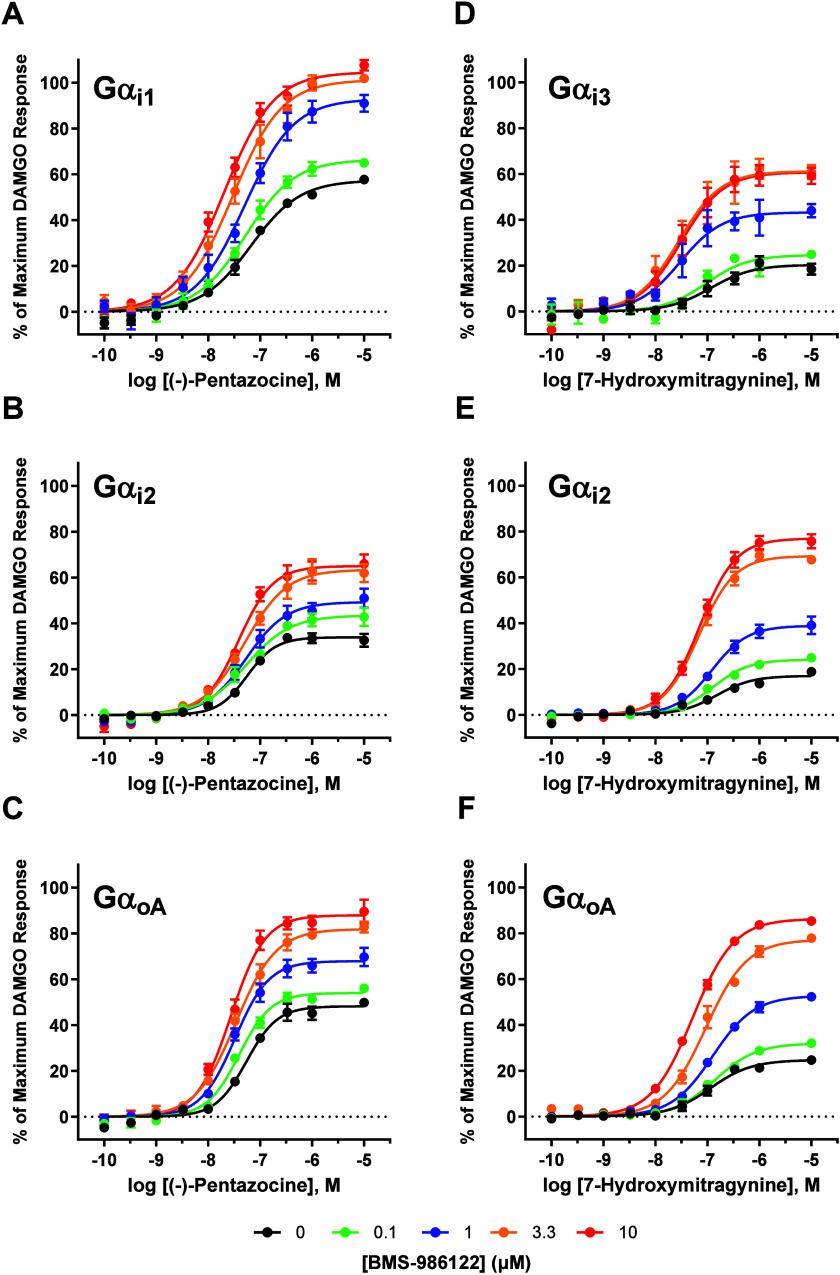
Effect of BMS-986122 on the potency (EC_50_) and efficacy (E_max_) of partial agonists when MOR signals through different Gα subunits. Concentration–response curves were generated for the two partial agonists (−)-pentazocine and 7-hydroxymitragynine using the nanoBRET assay in the presence of vehicle (●), 0.1 μM (green ●), 1.0 μM (blue ●), 3.3 μM (orange ●) and 10 μM (red ●) BMS-986122, when MOR signaled through Gαi1 (A), Gαi2 (B) and GαoA (C). BMS-986122 had a pronounced effect on the efficacy of the weak partial agonist 7-hydroxymitragnine, with the smallest effect observed when MOR signaled through Gαi3 (D), and a comparable effect between Gαi2 (E) and GαoA (F). All points are the mean values ± SEM from at least three independent experiments performed in duplicate.

**Table 5:** Relative Transduction Coefficients (Δlog­(τ)) of Partial Agonists between Treatment with Vehicle and 10 μM BMS-986122^a^

Subunit	(−)-Pentazocine Δlog(τ) ± SEM	7-Hydroxymitragynine Δlog(τ) ± SEM	Morphine Δlog(τ) ± SEM
Gαi1	0.865 ± 0.048	1.16 ± 0.10	0.693 ± 0.022
Gαi2	0.399 ± 0.043	1.31 ± 0.11	0.672 ± 0.055
Gαi3	0.442 ± 0.044	0.742 ± 0.087	0.754 ± 0.082
GαoA	0.492 ± 0.027	0.960 ± 0.038	0.612 ± 0.041
GαoB	0.568 ± 0.039	1.45 ± 0.082	0.755 ± 0.058
Gαz	0.239 ± 0.100	0.405 ± 0.092	0.057 ± 0.065

a Concentration–response data of partial agonists were fit to the operational model of agonism^33^ to derive relative transduction coefficients as described in Materials and Methods. Statistical analysis of differential efficacy modulation of partial agonists by BMS-986122 is shown in Figure S2. Data shown are the mean values ± SEM from at least three independent experiments performed in duplicate.

We next investigated the differential effects of BMS-986122 on 7-hydroxymitragynine, a weak partial agonist, on MOR signaling through distinct Gα subunits. Concentration–response curves were constructed for 7-hydroxymitragynine treated with vehicle and a range of PAM concentrations, up to 10 μM, when MOR signaled through Gαi3 (Figure 3D), Gαi2 (Figure 3E) and GαoA (Figure 3F). Differences in Gα subunit-specific efficacy modulation of 7-hydroxymitragynine by BMS-986122, quantified by Δlog­(τ) values, were the most pronounced than any of the agonists tested and reached statistical significance (Figure S2D). Again, BMS-986122 had very little effect on the efficacy of 7-hydroxymitragynine when MOR signaled through Gαz (Δlog­(τ) = 0.405 ± 0.092). The effect of BMS-986122 was relatively weak with Gαi3 (Δlog­(τ) = 0.742 ± 0.087) and was the most robust with GαoB (Δlog­(τ) = 1.45 ± 0.082). In order to ensure that the marked differences in efficacy modulation by BMS-986122 observed with 7-hydroxymitragynine was not a result of an assay artifact that is specific to the nanoBRET system, we tested the effects of BMS-986122 in an orthogonal BRET^2^ assay, TruPATH.^36^ The results from these experiments are shown in Figure S3. While the intrinsic efficacy of 7-hydroxymitragnine was often greater in the TruPATH assay compared to nanoBRET, the degree of efficacy modulation imparted by BMS-986122 when MOR signaled through GαoA (Figure S3A), GαoB (Figure S3B), and Gαi3 (Figure S3C), quantified by Δlog­(τ) values, was not statistically different when comparing the results obtained from the two assay systems (Figure S3D). These results suggest that the differential effects of BMS-986122 on 7-hydroxymitragynine are not dependent on the particular assay system used.

Given that 7-hydroxymitragynine is a very weak partial agonist at the MOR, we explored whether the intrinsic efficacy of the orthosteric agonist played a role in the capacity for differential efficacy modulation of BMS-986122 across different Gα subunits. To test this hypothesis, we constructed concentration–response curves with morphine, a high efficacy partial agonist, in the presence and absence of 10 μM BMS-986122 when MOR signaled through the six inhibitory Gα subunits (Figure S4). Remarkably, for morphine, the relative transduction coefficients for all subunits, except Gαz, were not statistically different from one another (Figure S4). This indicates that the G protein subtype-biased signaling imparted by BMS-986122 is more pronounced for agonists with low intrinsic efficacy and is diminished for both high efficacy partial agonists and full agonists.

### Apparent Affinity (K_B_) of BMS-986122 Increased Significantly When MOR Signaled through GαoB

Because we had collected concentration–response data for each agonist in the presence of multiple concentrations of BMS-986122 for a subset of Gα subunits, these data were amenable to fitting with the functional allosteric model^23,35^ via nonlinear regression. The functional allosteric model is a combination of the Black–Leff operational model and the allosteric two-state model developed by Ehlert.^45,46^ Fitting concentration–response data to this model yields system-independent parameters that quantify GPCR allosteric modulation: an affinity cooperativity factor (α), an efficacy cooperativity factor (β), and an affinity of the allosteric modulator (K_B_). A value of α or β greater than 1 indicates positive modulation of affinity or efficacy, respectively, while values less than 1 indicate negative allosteric modulation. The values of α calculated for full agonists, β values for partial agonists, and K_B_ values, when MOR signaled through distinct Gα subunits, are listed in Table 6. The relative α and β values obtained from this analysis for each Gα subunit were in good agreement with the potency ratios (ΔpEC_50_) and relative transduction coefficients (Δlog­(τ)) calculated for full and partial agonists, respectively.

**Table 6:** Functional Allosteric Model Parameters of MOR Full and Partial Agonists When MOR Signaled through Different Gα Subunits^a^

	DAMGO		Met-Enkephalin		(−)-Pentazocine		7-Hydroxymitragynine	
Subunit	α ± SEM	K_B_ (μM ± SEM)	α ± SEM	*K*_B_ (μM ± SEM)	β ± SEM	K_B_ (μM ± SEM)	β ± SEM	K_B_ (μM ± SEM)
Gαi1	5.0 ± 0.2	2.5 ± 0.06	6.9 ± 0.9	2.5 ± 0.6	10 ± 0.8	3.4 ± 1.2	ND	ND
Gαi2	5.9 ± 0.6	2.4 ± 0.9	3.2 ± 0.3	1.5 ± 0.6	3.3 ± 0.3	1.9 ± 0.03	10 ± 2.8	3.1 ± 0.7
Gαi3	ND	ND	ND	ND	ND	ND	5.9 ± 1.1	2.3 ± 0.8
GαoA	7.1 ± 1.0	1.6 ± 0.2	4.3 ± 0.7	3.5 ± 1.0	4.2 ± 0.3	2.5 ± 0.81	12 ± 1.8	8.0 ± 0.2
GαoB	ND	ND	ND	ND	4.2 ± 0.8	0.62 ± 0.18	15 ± 4.2	0.54 ± 0.10

a Concentration–response data of MOR full and partial agonists were fit to the functional allosteric model^23,35^ to derive allosteric parameters of BMS-986122 for select Gα subunits as described in Materials and Methods. Data shown are the mean values ± SEM from at least three independent experiments performed in duplicate.

A striking result from this analysis was that the apparent affinity (K_B_) estimate of BMS-986122 increased substantially when MOR was coupled to GαoB compared with the other Gα subunits tested. Figure 4 shows concentration–response curves for (−)-pentazocine (Figure 4A) and 7-hydroxymitragynine (Figure 4B) in the presence of a range of concentrations of BMS-986122 when MOR signaled through GαoB. Compared to the other Gα subunits tested, a 1 μM concentration of BMS-986122 was sufficient to achieve a saturating effect on agonist efficacy modulation for (−)-pentazocine and was nearly saturating for 7-hydroxymitragynine. Fitting these data to the functional allosteric model yielded a K_B_ of 0.62 ± 0.18 μM for (−)-pentazocine and 0.54 ± 0.10 μM for 7-hydroxymitragynine. For comparison, estimates of K_B_ for GαoA, a highly homologous subunit to GαoB were 2.5 ± 0.81 μM for (−)-pentazocine and 8.0 ± 0.2 μM for 7-hydroxymitragynine. To further explore this phenomenon, an R_50_ curve^35^ was generated, in which BMS-986122 was titrated in the presence of a constant concentration of agonist, in this case 10 μM 7-hydroxymitragyinine (Figure 4C). The R_50_ (the concentration of PAM which gives a half-maximal response) was 0.67 ± 0.11 μM, which is in good agreement to the K_B_ value calculated from the functional allosteric model. Interestingly, the Hill coefficient for this curve was calculated to be 3.0. Hill coefficients greater than 1.0 indicate binding cooperativity,^47,48^ which suggests that BMS-986122 is binding to the MOR cooperatively with GαoB.

**Figure 4:**
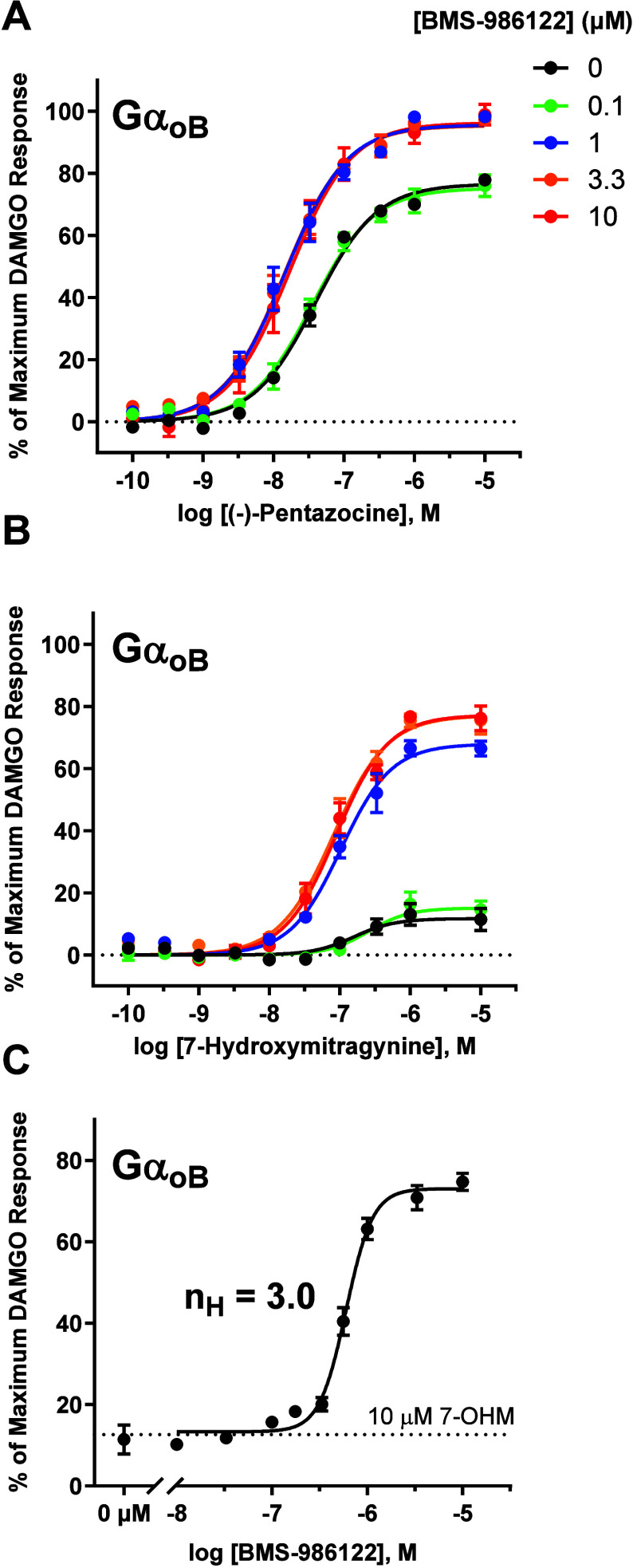
Apparent affinity (K_B_) of BMS-986122 for MOR increased substantially when MOR was coupled to GαoB. Concentration–response curves were generated for (−)-pentazocine (A) and 7-hydroxymitragynine (B) in the presence of vehicle (●), 0.1 μM (green ●), 1.0 μM (blue ●), 3.3 μM (orange ●) and 10 μM (red ●) BMS-986122 when MOR signaled through GαoB. (C) R_50_ curve^35^ of BMS-986122 with MOR signaling through GαoB, produced by pretreating cells with 10 μM 7-hydroxymitragynine and titrating BMS-986122. All points are the mean values ± SEM from at least three independent experiments performed in duplicate.

### BMS-986122 Acted as an Allosteric Agonist When MOR Signaled through Gαz, which Was Abolished by RGS20 Coexpression

To ensure that the effects on opioid signaling observed thus far for BMS-986122 were a result of either affinity or efficacy modulation alone and not due to the PAM having its own agonist effect (allosteric agonism), we collected concentration–response data of BMS-986122 when MOR signaled through the six inhibitory Gα subunits, Gαi1–3 (Figure S5A–C), GαoA (Figure S5D), GαoB (Figure S5E), and Gαz (Figure S5F). BMS-986122 up to a 10 μM concentration produced no response in the absence of any orthosteric agonist except when MOR signaled through Gαz, with an E_max_ value of 21 ± 2%. Considering that Gαz has a markedly slower intrinsic rate of GTP hydrolysis compared to the other inhibitory Gα subunits,^49^ we hypothesized that this unique feature of Gαz allowed it to strongly amplify a small degree of allosteric agonism intrinsic to BMS-986122. To test this, we collected concentration–response data of BMS-986122 in the absence and presence of RGS20 (RGSz1), a protein that catalyzes the hydrolysis of GTP to GDP for Gαz^50^ (Figure 5A). RGS20 co-expression was able to effectively eliminate the allosteric agonism of BMS-986122 when MOR signaled through Gαz.

**Figure 5:**
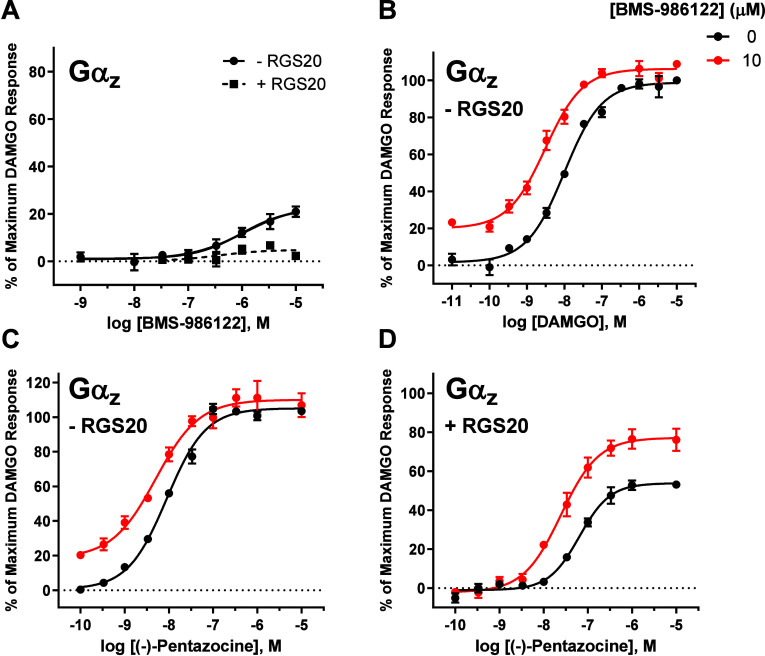
BMS-986122 acted as an allosteric agonist when MOR signaled through Gαz, which was abolished by RGS20 coexpression. (A) Concentration–response curves of BMS-986122 in the presence of vehicle (●) and 10 μM BMS-986122 (red ●), with and without RGS20 (RGSz1) coexpression when MOR signaled through Gαz. (B) The effect of BMS-986122 on the DAMGO concentration–response curve was observed when MOR signaled through Gαz in the absence of RGS20. (C) The effect of BMS-986122 on the (−)-pentazocine concentration–response curve when MOR signaled through Gαz in the absence of RGS20. (D) Concentration–response curves of (−)-pentazocine in the presence of vehicle (●) and 10 μM BMS-986122 (red ●) when MOR signaled through Gαz, with RGS20 coexpression. All points are the mean values ± SEM from at least three independent experiments performed in duplicate.

Concentration–response curves for DAMGO and (−)-pentazocine treated with either vehicle or 10 μM BMS-986122, when MOR signaled through Gαz, were additionally generated (Figure 5B,C). For both agonists, BMS-986122 modestly increased the potency and caused an upward shift of the curve due to the allosteric agonism of BMS-986122. When RGS20 was co-expressed, however, allosteric agonism again was lost. Interestingly, both the EC_50_ and the E_max_ value of (−)-pentazocine were dramatically reduced as well when RGS20 was coexpressed, which is also likely due to the acceleration of the rate of GTP hydrolysis by Gαz (Figure 5D). The coexpression of RGS20 likely reduces the pool of activated Gαz when steady state is achieved, which is reflected by a lowering of the activity of agonists. This phenomenon has also been observed for kappa opioid receptor agonists signaling through Gαz.^42^

### BMS-986122 and BMS-986187 Likely Bind to the Same Allosteric Site on MOR but Displayed Distinct G Protein Subtype Bias Factors

While the experiments reported in this paper focus primarily on the pharmacological properties of BMS-986122, there exist other allosteric modulators for opioid receptors, such as BMS-986187 and BMS-986124.^29,31^ BMS-986187 has been most extensively characterized at the delta opioid receptor, where it acts as a G protein-biased PAM-agonist.^29^ However, BMS-986187 also acts as a PAM for MOR agonists as well.^31^ BMS-986124 is a positional isomer of BMS-986122 and is known as a “silent allosteric modulator” due to it having no PAM effect on its own but has been shown to competitively antagonize the action of BMS-986122 on MOR agonists.^24^

Initially, we sought to confirm whether the binding site of BMS-986187 and BMS-986122 on the MOR is shared. Figure 6A shows concentration–response curves of DAMGO when MOR signaled through Gαi2, with cells treated with vehicle, 10 μM BMS-986122, 10 μM BMS-986187, and 10 μM each PAM simultaneously. If both PAMs bound to the MOR at separate allosteric sites, we would anticipate an additive effect on the potency of DAMGO if the cells are treated with both simultaneously. However, we did not observe any additive effect, indicating that both PAMs are likely binding to the same site. To ensure this, we conducted an additional set of experiments in which the effect of 3 μM BMS-986122 or 3 μM BMS-986187 on the potency of DAMGO was challenged by the simultaneous addition of 10 μM BMS-986124 (Figures 6B and 6C, respectively). As observed in previous work,^24^ 10 μM BMS-986124 antagonized the effect of 3 μM BMS-986122, but not completely. While the binding site of BMS-986122 was recently elucidated by cryogenic electron microscopy,^51^ there presently exists no structural data that reveals the binding site of BMS-986124. However, given that it is a positional isomer of BMS-986122, it is likely that it is binding to the same allosteric site as BMS-986122 and is acting as a competitive antagonist. When this experiment was repeated for BMS-986187, we observed that a 10 μM concentration of BMS-986124 was also able to partially block the effect of 3 μM BMS-986187 (Figure 6C), similar to what was observed for BMS-986122. These results suggest that BMS-986122, BMS-986124, and BMS-986187 likely all bind to the same allosteric site on the MOR.

**Figure 6:**
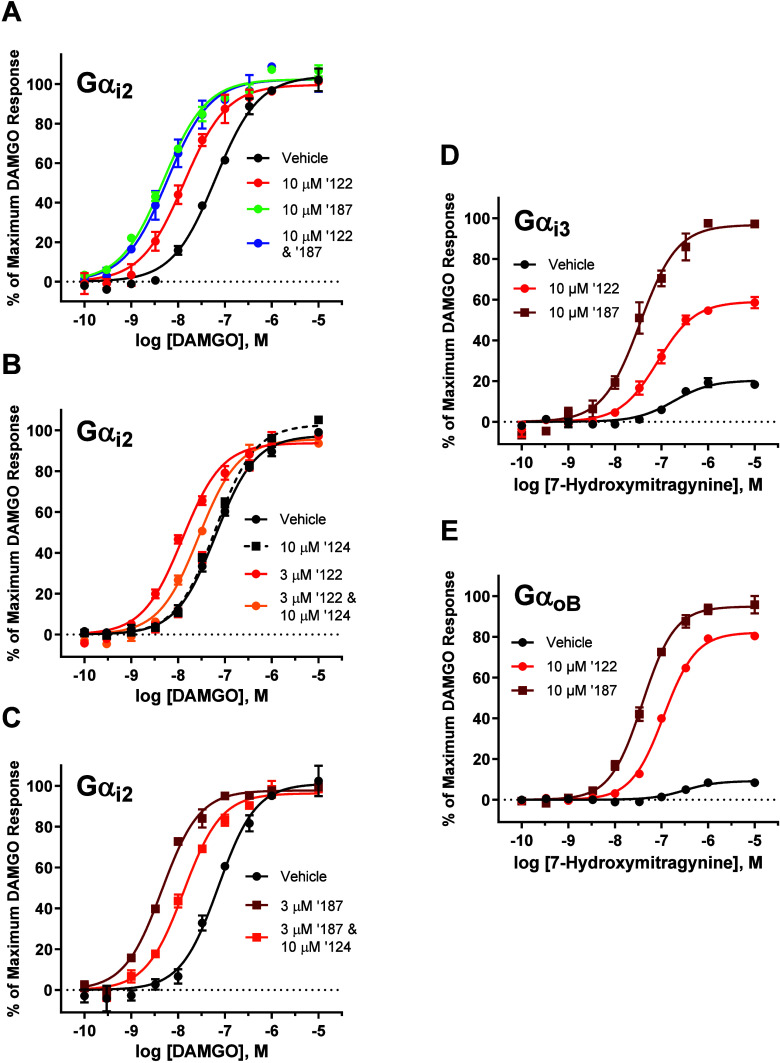
Comparison of the effects of BMS-986122 and BMS-986187 on MOR signaling through different Gα subunits. (A) Concentration–response curves of DAMGO in the presence of vehicle (●), 10 μM BMS-986122 (′122) (red ●), 10 μM BMS-986187 (′187) (green ●), and 10 μM both ′122 and ′187 (blue ●). (B) Concentration–response curves of DAMGO in the presence of vehicle (●), BMS-986124 (′124) (■), 3 μM ′122 alone (red ●) and 3 μM ′122 and 10 μM ′124 simultaneously (orange ●). (C) Concentration–response curves of DAMGO in the presence of vehicle (●), 3 μM ′187 alone (red ■) and 3 μM ′187 and 10 μM ′124 simultaneously (orange ■). Concentration–response curves for 7-hydroxymitragnine in the presence of vehicle (●), 10 μM ′122 (red ●), and 10 μM ′187 (red ■) when MOR signaled through Gαi3 (D) and GαoB (E). All points are the mean values ± SEM from at least three independent experiments performed in duplicate.

Given these results, we sought to determine whether BMS-986187 would exhibit a similar G protein subtype bias profile for MOR agonists compared to that of BMS-986122. We constructed concentration–response curves of 7-hydroxymitragnine in the absence and presence of 10 μM BMS-986187 when MOR signaled through Gαi3 (Figure 6D) and GαoB (Figure 6E). As observed previously, BMS-986122 displayed a diminished ability to modulate the efficacy of 7-hydroxymitragnine when MOR signaled through Gαi3 compared to GαoB, where efficacy modulation was the highest. Comparing the relative transduction coefficients between Gαi3 and GαoB for 7-hydroxymitragnine, ΔΔlog­(τ)_oB‑i3_ = 0.708. Strikingly, we did not observe the same differential effect on the efficacy of 7-hydroxymitragnine between Gαi3 and GαoB for BMS-986187, giving a ΔΔlog­(τ)_oB‑i3_ value of 0.157. Therefore, although BMS-986122 and BMS-986187 likely bind to the same allosteric site on the MOR, they displayed distinct PAM bias profiles for different Gα protein subtypes.

### Site-Directed Mutagenesis Studies Revealed Key Amino Acid Residues Involved in G Protein Signaling Bias of BMS-986122

Having observed such robust differences in the ability of BMS-986122 to increase the efficacy of 7-hydroxymitragnine depending on which Gα subunit MOR signaled through, we considered the possibility that amino acid residue differences in the C-termini of the inhibitory Gα proteins was involved in this effect. The C-terminus of all Gα proteins adopt an alpha helical shape and is the major structural element of G protein binding to a GPCR within the intracellular vestibule.^52,53^ Additionally, residue differences in the C-termini of Gα proteins have been shown to control the selectivity in the ability of GPCRs to only activate a specific family of G proteins, such as Gαs vs Gαq/11.^54,55^ Figure 7A shows a sequence alignment of the last 18 amino acids of all inhibitory Gα subunits, and relevant differences are color coded. One of the major differences between Gαo and Gαi subunits is at position 350, where Gαi subunits contain a negatively charged amino acid residue (aspartate for Gαi1–2 and glutamate for Gαi3), while Gαo subunits contain a neutral glycine residue. Additionally, Gαi1–3 subunits as well as Gαz contain a lysine at position 349 while Gαo subunits have an arginine.

**Figure 7:**
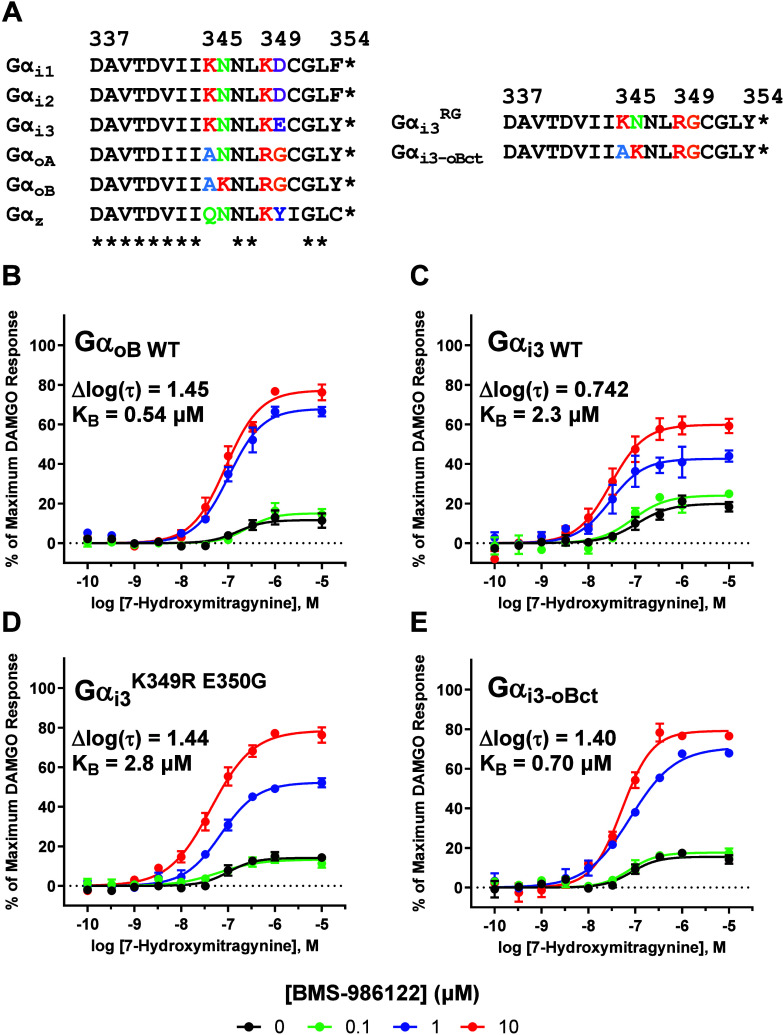
Effect of Gαi3 C-terminal amino acid mutations on the allosteric action of BMS-986122 on 7-hydroxymitragynine signaling. (A) Sequence alignment of the last 18 amino acids of all human inhibitory Gα proteins and sequences of two Gαi3 C-terminal mutants generated. Concentration–response curves of 7-hydroxymitragnine in the presence of vehicle (●) 0.1 μM (green ●), 1.0 μM (blue ●), and 10 μM (red ●) BMS-986122 when MOR signaled through WT GαoB (B) and WT Gαi3 (C). (D) Concentration–response curves of 7-hydroxymitragnine in the presence of varying concentrations of BMS-986122 when MOR signaled through a mutant of Gαi3 where K349 and E350 were substituted for R and G, respectively (Gαi3^K349R, E350G^). (E) Concentration–response curves of 7-hydroxymitragnine in the presence of varying concentrations of BMS-986122 when MOR signaled through a mutant of Gαi3 in which the C-terminus was completely replaced by the C-terminus of GαoB. All plotted points are the mean values ± SEM from at least three independent experiments performed in duplicate. Model parameter estimates are the mean values from at least three independent experiments performed in duplicate.

Focusing our initial mutagenesis experiments on the residues at positions 349 and 350, we created a mutant of Gαi3 in which K349 and E350 were mutated to R and G, respectively (Gαi3^K349R, E350G^), thus mimicking the amino acids observed in Gαo at those positions. We constructed concentration–response curves of 7-hydroxymitragnine in the absence and presence of a range of concentrations of BMS-986122 when MOR signaled through the Gαi3^K349R, E350G^ mutant and directly compared the results to the curves obtained when MOR signaled through WT GαoB (Figure 7B) and WT Gαi3 (Figure 7C), shown in Figure 7D. After fitting to the operational model of agonism, we calculated a Δlog­(τ) value of 1.44 ± 0.09 for the mutant Gαi3, and fitting to the functional allosteric model yielded a K_B_ value of 2.8 ± 0.4 μM for BMS-986122. This suggests that R349 and/or G350 are important residues on Gαo that allow for a greater effect by BMS-986122 on the efficacy of 7-hydroxymitragnine. Given that the K_B_ value of BMS-986122 for the mutant was not statistically different than the K_B_ value obtained when MOR signaled through WT Gαi3 (2.3 ± 0.8 μM), it is unlikely that these residues are involved in the affinity increase of BMS-986122 observed when MOR signaled through GαoB.

Attempting to completely recapitulate the pharmacological properties of BMS-986122 when MOR signaled through GαoB, two additional mutations on the Gαi3^K349R, E350G^ construct were made, mutating K345 to an alanine and N346 to a lysine residue. These two mutations effectively create a chimera between Gαi3 and GαoB, in which the C-terminus of Gαi3 is replaced with that of GαoB (Gαi3-oBct). We ran the Gαi3-oBct mutant in the nanoBRET assay with 7-hydroxymitragnine as the agonist and included a range of concentrations of BMS-986122, with the results shown in Figure 7E. Here, we calculated a Δlog­(τ) value of 1.40 ± 0.04, which was not significantly different from the Δlog­(τ) values obtained for both WT GαoB (1.45 ± 0.082) and Gαi3^K349R, E350G^ (1.44 ± 0.09). Importantly, however, fitting these data to the functional allosteric model yielded a K_B_ of 0.70 ± 0.06 μM, which was not statistically different from the K_B_ value of 0.54 ± 0.10 μM obtained for BMS-986122 when MOR signaled through WT GαoB.

An examination of the sequences of inhibitory Gα subunit C-termini reveals a single amino acid that is exclusive to GαoB, K346, where all other inhibitory Gα subunits contain an asparagine residue. Considering the possibility that this residue is involved in the increase in affinity of BMS-986122 when MOR signaled through GαoB, we created a mutant of Gαi2 in which the asparagine residue at position 346 was mutated to a lysine (Gαi2^N346 K^), mimicking GαoB at that position. The results obtained from running WT Gαi2 in the nanoBRET assay with 7-hydroxymitragnine as the agonist in the absence and presence of a range of concentrations of BMS-986122 are shown in Figure 8A. Comparing these results to those obtained with the Gαi2^N346 K^ mutant (Figure 8B) reveals that, with the Gαi2 mutant, a 1 μM concentration of BMS-986122 was nearly sufficient in achieving a saturating effect on efficacy modulation, similar to what was observed with WT GαoB. Fitting these data to the functional allosteric model yielded a K_B_ value of 0.69 ± 0.06 μM, which was not statistically different than the K_B_ value of 0.54 ± 0.10 μM calculated for WT GαoB. Overall, these results indicate that there exist molecular determinants for G protein subtype-biased signaling imparted by BMS-986122 within amino acid residue differences across the C-termini of inhibitory Gα subunits.

**Figure 8:**
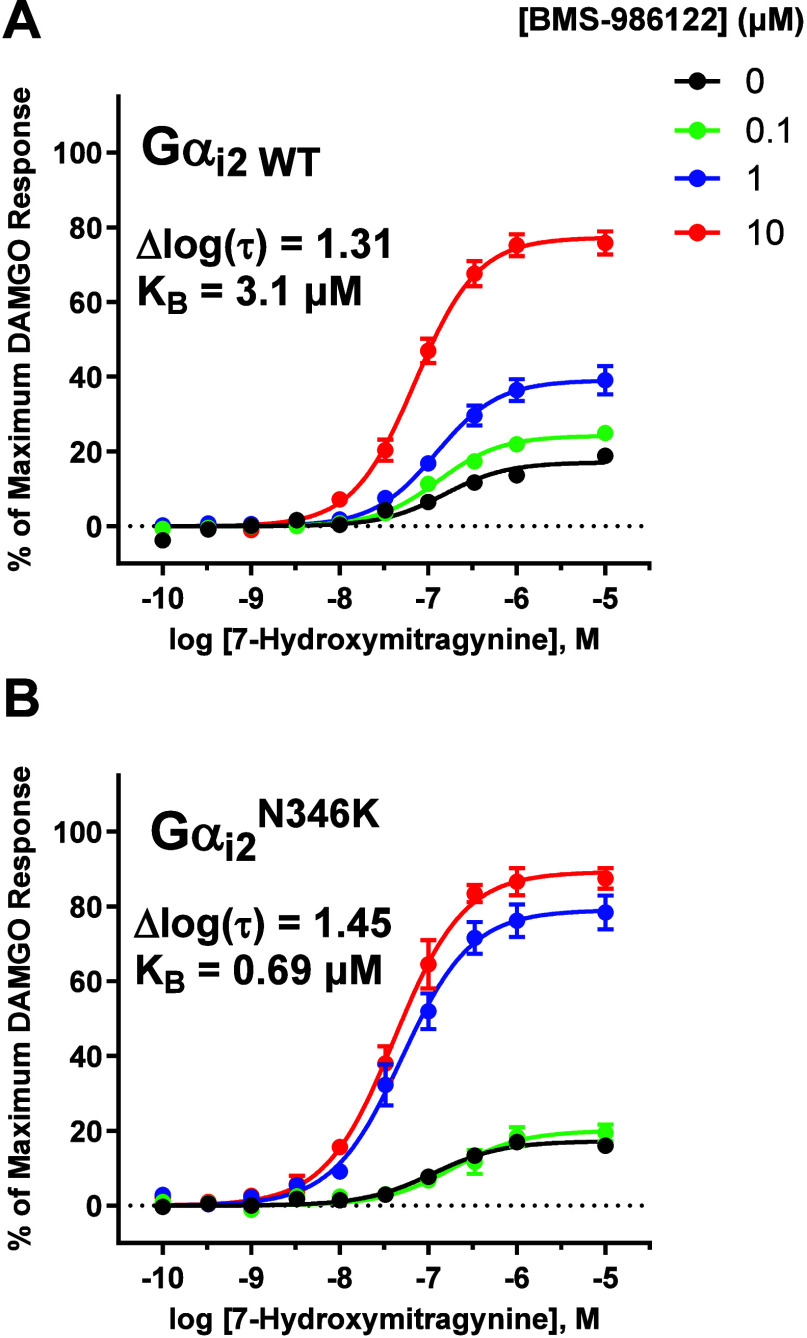
K346 on GαoB is responsible for the increased affinity of BMS-986122 when the MOR is coupled to GαoB. (A) Concentration–response curves of 7-hydroxymitragynine in the presence of vehicle (●) 0.1 μM (green ●), 1.0 μM (blue ●), and 10 μM (red ●) BMS-986122 when MOR signaled through WT Gαi2. (B) Concentration–response curves of 7-hydroxymitragnine in the presence of varying concentrations of BMS-986122 when MOR signaled through a mutant of Gαi2 where N346 was substituted for K, mimicking GαoB at that position. All plotted points are the mean values ± SEM from at least three independent experiments performed in duplicate. Model parameter estimates are the mean values from at least three independent experiments performed in duplicate.

## Discussion

A nanoBRET-based functional assay system was utilized in live HEK 293T cells to determine the unique pharmacology of the MOR PAM BMS-986122 when MOR signaled through different Gα subunits. The initial competition binding experiments revealed that BMS-986122 increased the affinity of full agonists DAMGO and met-enkephalin and had no effect on the binding of partial agonists (−)-pentazocine and 7-hydroxymitragnine. The capacity for allosteric modulators to differentially modulate the affinity and/or efficacy of distinct orthosteric agonists is termed probe dependence.^23,56^ In other studies,^24,30^ BMS-986122 was shown to display probe dependence defined by the intrinsic efficacy of the orthosteric agonist, where full agonist affinity was increased without any further increase in efficacy, and partial agonist efficacy was increased with no change in affinity. This feature of BMS-986122 was explained in part due to its ability to disrupt the binding of allosteric sodium to the receptor once bound.^30^ The binding affinity of MOR full agonists are highly sensitive to the concentration of Na^+^ ions, exhibiting a lower affinity in the presence of high [Na^+^], while partial agonists are relatively insensitive to the presence of Na^+^ ions.^57–59^ Therefore, the disruption of Na^+^ ion binding to the MOR by the binding of BMS-986122 had no effect on the affinity of the partial agonists.

The overall results from the nanoBRET assay experiments indicate that BMS-986122 differentially modulates the signaling properties of opioids depending on which Gα subunit MOR signals through. Notably, however, this effect appears to be highly dependent on the orthosteric agonist cobinding to the receptor. With met-enkephalin, BMS-986122 produced only modest Gα subunit bias toward Gαi1 activation, and with DAMGO BMS-986122 displayed no statistically significant differences of potency modulation between the six Gα subunits. These results suggest that attempts to bias MOR signaling toward the recruitment of specific G protein subtypes using allosteric modulators may prove difficult when considering only affinity modulation with full agonists. Additionally, while G protein bias was observed for partial agonists (−)-pentazocine and 7-hydroxymitragnine, the high efficacy partial agonist morphine was unable to be biased toward specific G protein subtypes by BMS-986122, except for being slightly biased away from Gαz activation. It is conceivable that BMS-986122 is capable of imparting G protein subtype bias to lower efficacy partial agonists and that this effect begins to diminish as the intrinsic efficacy of the orthosteric agonist increases. In order to validate this, however, additional experiments must be performed with other low efficacy partial agonists other than (−)-pentazocine and 7-hydroxymitragnine. If it is the case that BMS-986122 imparts a stronger G protein subtype bias to weak partial agonists generally, this may end up being highly useful if future novel allosteric modulators can preserve this feature. Beginning from a low, relatively unbiased background of G protein activation by the orthosteric agonist alone would allow for the selection of an individual or a subset of G protein subtypes through selective efficacy enhancement by a PAM, resulting in a much stronger signaling bias. While it is unclear which inhibitory Gα proteins mediate the specific biological actions of opioids, the development of biased allosteric modulators that effectively select a single Gα protein by specifically enhancing opioid signaling through it would be an extremely useful tool to determine which Gα proteins are necessary for particular opioid effects *in vivo*.

Regarding the overall findings of the unique G protein signaling properties of BMS-986122, a few trends in the modulation by the PAM may be noted: generally, Gαi1 and GαoB activation by MOR agonists appeared to be strongly improved, while Gαi3 and Gαz activation were comparatively improved only weakly by the PAM. It is unclear whether the uniquely low enhancement of Gαz signaling by BMS-986122 is connected to the allosteric agonism observed when MOR signaled through Gαz, though this may very well be the case. Additionally, given the results obtained from the comparison of the effects of BMS-986122 and BMS-986187 on the modulation of 7-hydroxymitragnine, it is unlikely that the trends observed for BMS-986122 in this work will be identical, or even similar, to those of other allosteric modulators of the MOR. Future experiments that include an extensive characterization of the potential G protein subtype bias imparted by BMS-986187, as well as other PAMs for the MOR, may be very useful for the determination of structure–activity relationships involved in biased allosteric modulation.

Fitting our data to the operational model allowed for a rapid and relatively straightforward means of quantifying our results through the calculation of relative transduction coefficients (Δlog­(τ)) for partial agonists compared between each Gα subunit that MOR signaled through. It is quite promising for the rigor of this analysis that the comparison between Δlog­(τ) values obtained for 7-hydroxymitragnine between nanoBRET and TruPATH assay platforms yielded nearly identical results, indicating that these values are system-independent.^34^ In comparison, fitting concentration–response data to the functional allosteric model was often cumbersome, as both high-quality data relatively free of noise and multiple PAM concentrations were necessary to obtain an adequate fit for the estimation of model parameters. It is important to note, however, that in the original study of MOR PAMs conducted by Burford et al.,^24^ the affinity cooperativity factor α for BMS-986122 was calculated to be 7.0 and K_B_ was calculated to be 5.0 μM when endomorphin-1 was the orthosteric agonist through fitting to the allosteric ternary complex model. These estimates are similar in value to the results obtained for full agonists DAMGO and met-enkephalin in this study, suggesting consistency in this type of analysis. Moreover, a recent publication by Krumm et al.^21^ utilized the functional allosteric model to quantify the properties of the neurotensin receptor 1 AM SBI-553, which was found to be a highly complex-biased allosteric modulator when assessing multiple G protein signaling pathways (Gαi/o, Gαq, and others), as well as β-arrestin recruitment. Because SBI-553 exhibited either positive or negative allosteric effects on agonist affinity/efficacy, as well as differing degrees of allosteric agonism, depending on which transducer protein the receptor signaled through, estimations of parameters such as α, β, K_B_, and τ_B_ (efficacy of the AM), were necessary to exhaustively characterize the complex behavior of the AM.

A useful result of fitting our data to the functional allosteric model that could not be achieved through analysis with the operational model alone was the estimation of the binding affinity (K_B_) of BMS-986122, which led to the discovery that GαoB selectively enhanced the binding of BMS-986122 compared with the other Gα subunits tested. This discovery is highly relevant to the calculation of bias factors for allosteric modulators, as it is often the case that only a comparison of agonist activity between a treatment with a saturating PAM concentration and a vehicle treatment is taken. In a publication by Kenakin,^44^ the term Δlog­(αβ/K_B_) was proposed as a means of calculating overall changes in allosteric action that, when applied to differential modulation between two signaling pathways, would effectively incorporate PAM affinity into the calculation of allosteric bias. Interestingly, the BRET results observed in this study for GαoB suggest that BMS-986122 will strongly bias opioid signaling toward GαoB activation at subsaturating concentrations, and this effect will diminish as BMS-986122 reaches saturating concentrations. Future experiments will be necessary to determine not only whether other MOR PAMs, such as BMS-986187, exhibit this property but also if it is conserved across different opioid receptors as well.

Lastly, site-directed mutagenesis studies with Gαi3 were highly useful as an initial point of departure for the determination of the molecular mechanisms of differential efficacy modulation by BMS-986122 as well as the increase in PAM affinity observed when MOR signaled through GαoB. Two C-terminal amino acid residues were mutated for Gαi3 (K349R E350G), which produced a mutant that more closely resembled GαoB in terms of the ability of BMS-986122 to enhance the efficacy of 7-hydroxymitragnine. While mutants that carried only an individual mutation at either position 349 or 350 were not produced, it is likely that the E350G mutation was responsible for the shift from Gαi3 to GαoB character, given that this mutation neutralized a negative charge and the K349R mutation preserved the positive charge at that position. Recently, Kaneko et al.^51^ solved the structure of the MOR in complex with DAMGO, Gαi3, and BMS-986122 using cryoEM, with the proposed binding site of BMS-986122 being a pocket formed by transmembrane helices 3, 4, and 5, above the second intracellular loop (ICL2). It is interesting to note that E350 on Gαi3 interacts with ICL2 by forming a salt bridge with R181 on the MOR. This interaction would not be present on Gαo proteins that contain a glycine residue at this position. It is possible that differential interactions with the C-termini of Gα subunits and ICL2 of the MOR contribute to the differences in the capacity of BMS-986122 to enhance the efficacy of 7-hydroxymitragnine once bound, especially because ICL2 contains the E/DRY motif, which is involved in the activation mechanism of all class A GPCRs.^60–62^ Nevertheless, more experiments must be performed to conclusively verify these considerations.

A few caveats exist with site-directed mutagenesis studies. First, it is likely that differences in the C-termini of Gα are not solely responsible for differential efficacy modulation by BMS-986122 when MOR signals through different Gα subunits. BMS-986122 enhanced the signaling of (−)-pentazocine and met-enkephalin the most when MOR signaled through Gαi1. Given that the C-termini of Gαi1 and Gαi2 are identical, the capacity for BMS-986122 to better enhance Gαi1 signaling for these agonists must be due to some other structural difference between Gαi1 and the other Gα subunits outside of the C-terminus. Another caveat is that while the production of mutants and chimeras were undoubtedly useful in determining specific amino acid residues important for differential efficacy modulation by BMS-986122, it does not always yield anticipated results, as the mutant protein can exhibit emergent properties that resemble neither the wild-type nor the protein that it is trying to mimic. For instance, an attempt was made to recapitulate the effect of BMS-986122 on 7-hydroxymitragnine when MOR signaled through Gαi3 by producing a chimera in which the C-terminus of GαoB was swapped for Gαi3 (GαoB-i3ct). While the efficacy modulation by BMS-986122 was reduced when MOR signaled through this construct, the intrinsic efficacy of 7-hydroxymitragnine unexpectedly increased substantially (data not shown). It is therefore important not to rely heavily on site-directed mutagenesis studies to determine overall molecular mechanisms.

## Conclusions

In summary, our findings illustrate that the MOR PAM BMS-986122 produces unique allosteric effects on different opioid agonists depending on which inhibitory Gα subunit the MOR signals through. BMS-986122 increased only the affinity of full agonists and only the efficacy of partial agonists and differentially enhanced the efficacy of weak partial agonists 7-hydroxymitragnine and (−)-pentazocine when MOR signaled through different Gα subunits. Additionally, BMS-986122 was an allosteric agonist only when MOR signaled through Gαz. The Gα subtype signaling bias imparted by BMS-986122 was more pronounced for weak partial agonists and was diminished or nonexistent for high efficacy partial agonists and full agonists, such as morphine and DAMGO, respectively. Moreover, the binding affinity of BMS-986122 increased significantly only when MOR signaled through GαoB. Lastly, results obtained from site-directed mutagenesis studies suggest that the Gα subunit-specific modulation of BMS-986122 on opioid signaling can be partly explained by a small number of amino acid residue differences between the C-termini of Gαi/o subunits, which forms the α5 helix, a major recognition site on Gα proteins for GPCR binding. It is our hope that the results obtained in this study may provide guidance for the further development of MOR allosteric modulators and a deeper understanding of the molecular mechanisms of GPCR-biased signaling.

## Supplementary Material



## Notes

Schematic of nanoBRET assay system; additional nanoBRET experiments including concentration–response data of BMS-986122 alone for all inhibitory Gα subunits and morphine concentration–response curves in the presence and absence of BMS-986122 for all inhibitory Gα subunits; comparison between nanoBRET and TruPATH assays for the effects of BMS-986122 on 7-hydroxymitragnine signaling through Gαi3, GαoA, and GαoB; and statistical analysis of BMS-986122 allosteric bias for different Gα subunits (PDF)

## Notes

The UniProt accession code IDs for MOR, Gαi1, Gαi2, Gαi3, Gαo, Gαz are as follows: P35372, P63096, P04899, P08754, P09471, P19086.

## Abbreviations

MOR: mu opioid receptor
GPCR: G protein-coupled receptor
PAM: positive allosteric modulator
AM: allosteric modulator
BRET: bioluminescence resonance energy transfer
DAMGO: ([d-Ala 2, N-MePhe 4, Gly-ol]-enkephalin)
MOR-CHO: Chinese hamster ovary cells stably expressing the human mu opioid receptor
HEK 293T: human embryonic kidney 293T cells
ICL2: intracellular loop 2
