# Supplementary material for: Mu Opioid Receptor Positive Allosteric Modulator BMS-986122 Confers Agonist-Dependent G Protein Subtype Signaling Bias
Source: Biochemistry. 2025 May 16;64(11):2376–93. doi: 10.1021/acs.biochem.5c00022 (PMC12138978; doi:10.1021/acs.biochem.5c00022)
Supplement: Supplementary file 1 [file bi5c00022_si_001.pdf]

Supporting Information

Mu Opioid Receptor Positive Allosteric Modulator BMS-986122 Confers Agonist-Dependent G Protein Subtype Signaling Bias

*Grant M. Grieble[1], Brian I. Knapp[1], and Jean M. Bidlack[1]**

[1] Department of Pharmacology & Physiology, University of Rochester School of Medicine and Dentistry, Rochester, NY 14642, USA

[*] Correspondence to: Jean M. Bidlack, Department of Pharmacology and Physiology, P.O. Box 711, University of Rochester School of Medicine and Dentistry, Rochester, New York 14642-8711, USA; Tel. (585) 275-5600; Fax (585) 273-2652; E-Mail: Jean_Bidlack@urmc.rochester.edu

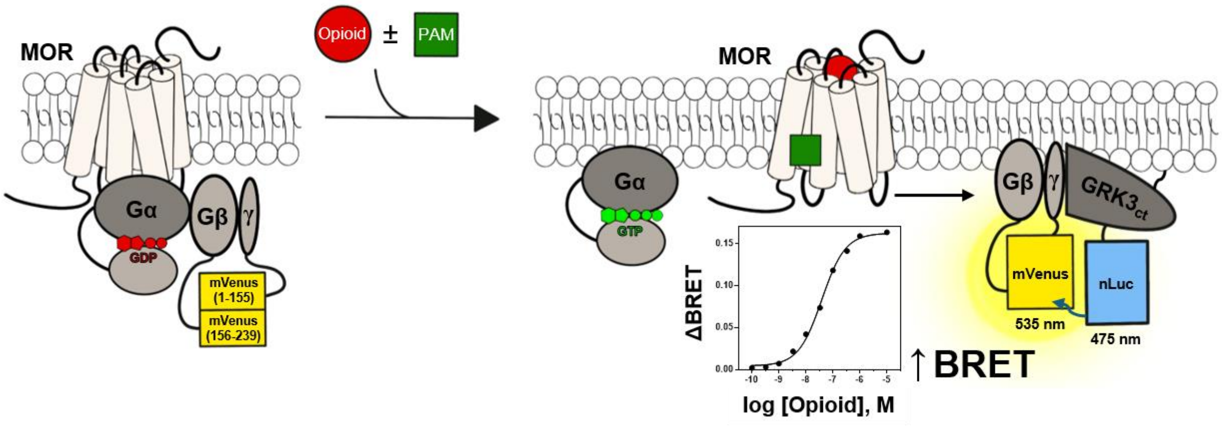

Figure S1. Overview of the nanoBRET functional assay. HEK 293T cells are transiently transfected with plasmids encoding for the human MOR (hMOR), Gα subunit of interest, $G\beta_1$-mVenus(156-239), $G\gamma_2$-mVenus(1-155), and the truncated C-terminus of GRK3 (a downstream effector of Gβγ) with myristic acid attachment sequence, fused to nanoluciferase (masGRK$_{ct}$-nLuc). The BRET acceptor (mVenus) is split between $G\beta_1$ and $G\gamma_2$ and is thus only functional when the two subunits form the obligate dimer. Upon agonist stimulation of MOR, Gα exchanges GDP for GTP, allowing for Gβγ-mVenus to dissociate from Gα and bind to masGRKct-nLuc, bringing mVenus in proximity to the BRET donor, nLuc. In the presence of its substrate furimazine, nLuc emits light at a wavelength of 475 nm. However, when in proximity to mVenus, resonance energy transfer may occur instead, where nLuc directly excites the chromophore of mVenus which then emits light at 535 nm. Both acceptor and donor emissions at 535 nm and 475 nm, respectively, are detected by a microplate reader and the BRET ratio (ratio of acceptor to donor emissions) is calculated, with an increase in the BRET ratio being correlated to the activation of Gα through agonist-bound MOR.

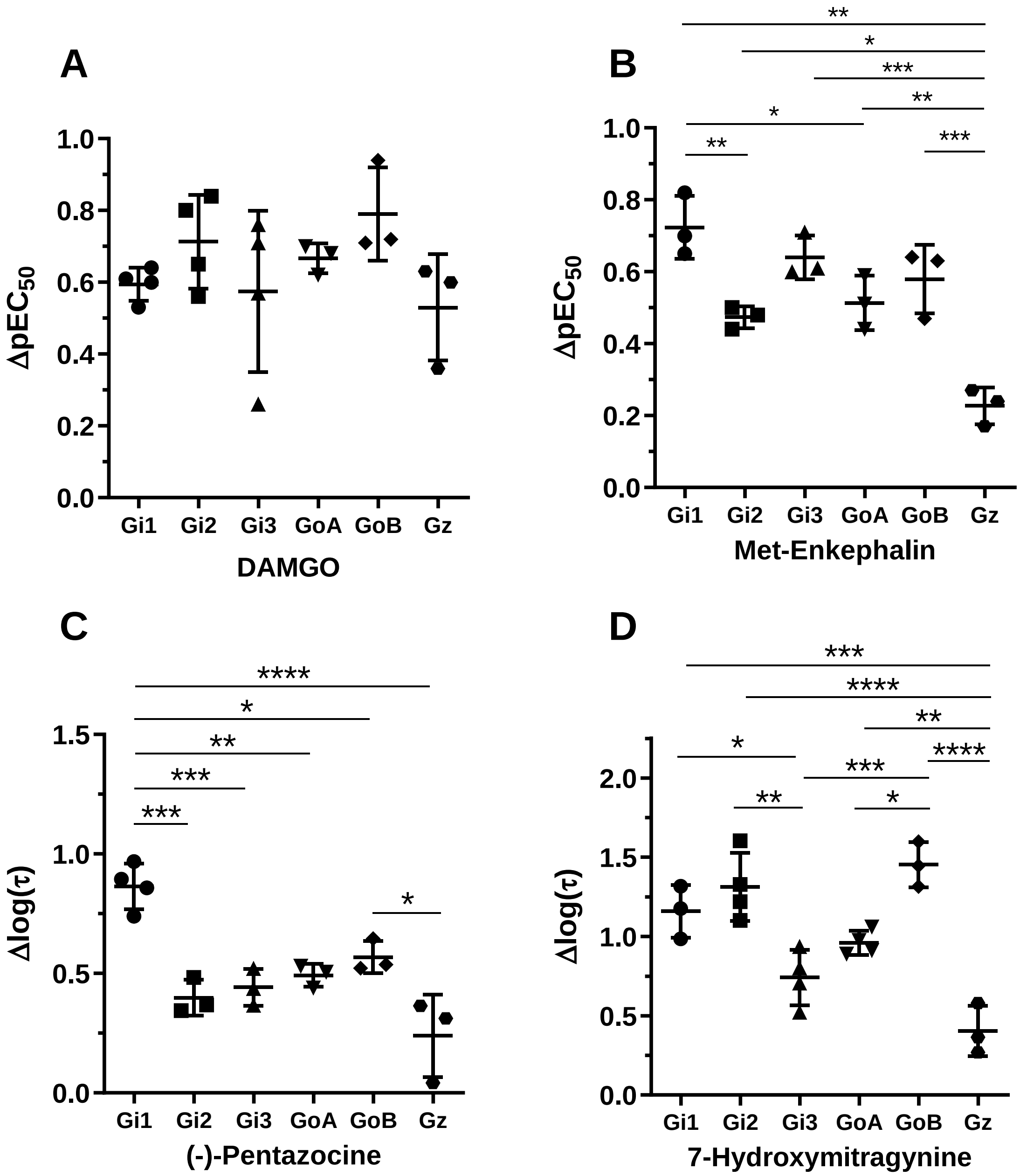
A
ΔpEC50
1.0
0.8
0.6
0.4
0.2
0.0
Gi1 Gi2 Gi3 GoA GoB Gz
DAMGO
B
**
*
***
**
*
**
***
ΔpEC50
1.0
0.8
0.6
0.4
0.2
0.0
Gi1 Gi2 Gi3 GoA GoB Gz
Met-Enkephalin
C
****
*
**
***
***
*
Δlog(τ)
1.5
1.0
0.5
0.0
Gi1 Gi2 Gi3 GoA GoB Gz
(-)-Pentazocine
D
***
****
**
*
****
***
**
*
Δlog(τ)
2.0
1.5
1.0
0.5
0.0
Gi1 Gi2 Gi3 GoA GoB Gz
7-Hydroxymitragynine

Figure S2. BMS-986122 allosteric bias plots of MOR full and partial agonists for all Gα subunits. **(A)** Potency ratios between DAMGO with vehicle pretreatment and DAMGO with 10 μM BMS-986122 when MOR signaled through different Gα subunits. No statistically significant difference in the ability of BMS-986122 to modulate the potency of DAMGO was observed between the six Gα subunits. **(B)** Potency ratios between met-enkephalin with vehicle pretreatment and met-enkephalin with 10 μM BMS-986122 when MOR signaled through different Gα subunits. **(C)** Relative transduction coefficients between (-)-pentazocine with vehicle treatment and 10 μM BMS-986122 treatment when MOR signaled through different Gα subunits. **(D)** Relative transduction coefficients between 7-hydroxymitragynine with vehicle pretreatment 10 μM BMS-986122 pretreatment when MOR signaled through different Gα subunits. * denotes $p<0.05$, ** $p<0.01$, *** $p<0.001$, **** $p<0.0001$, one-way ANOVA with Tukey's post-hoc test. All points are the mean values ± SD from at least three independent experiments performed in duplicate.

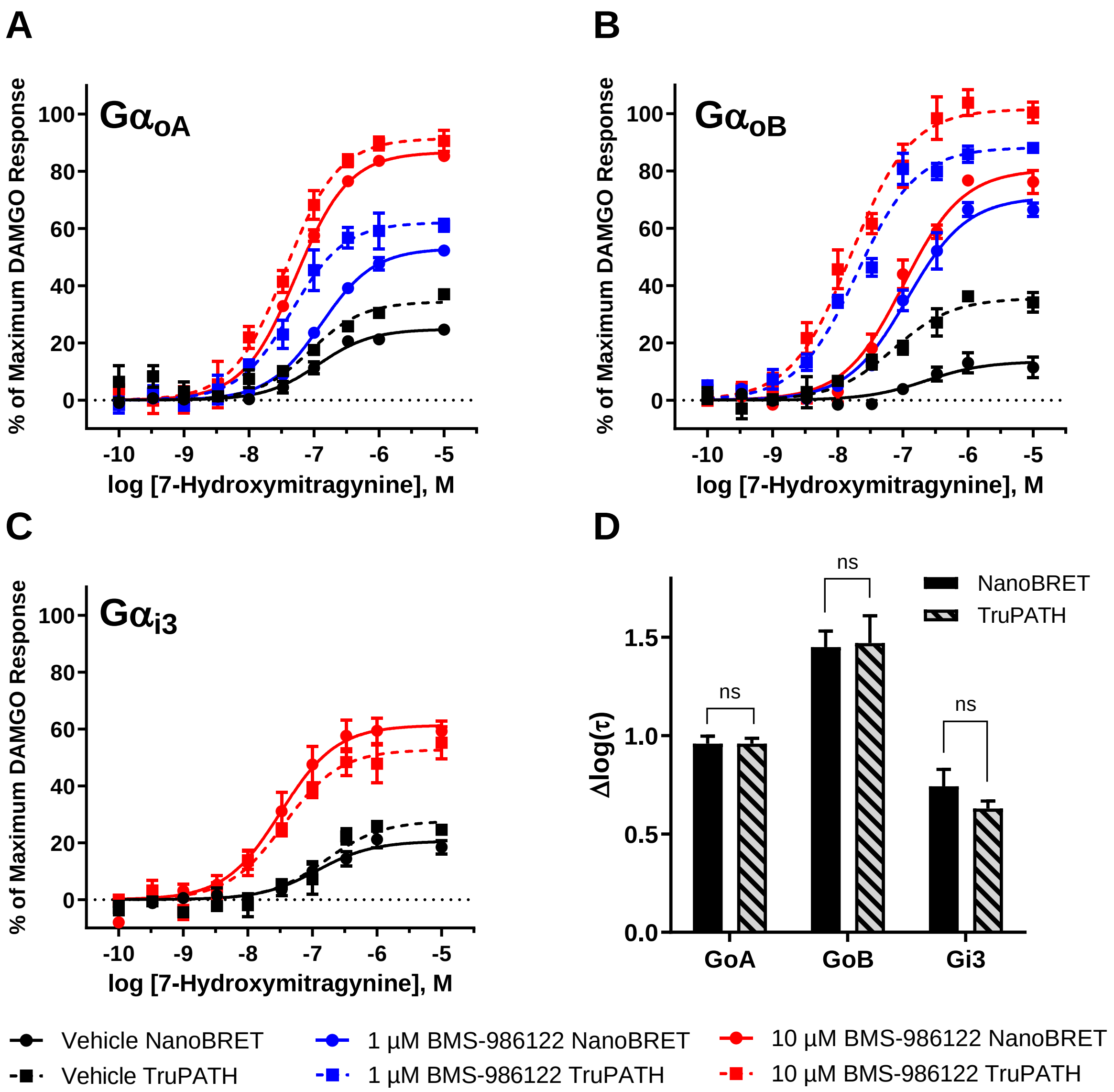
A
B
C
D
GαoA
GαoB
Gαi3
% of Maximum DAMGO Response
log [7-Hydroxymitragynine], M
100
80
60
40
20
0
-10
-9
-8
-7
-6
-5
Δlog(τ)
1.5
1.0
0.5
0.0
ns
NanoBRET
TruPATH
GoA
GoB
Gi3
Vehicle NanoBRET
Vehicle TruPATH
1 µM BMS-986122 NanoBRET
1 µM BMS-986122 TruPATH
10 µM BMS-986122 NanoBRET
10 µM BMS-986122 TruPATH

Figure S3. Comparison of the effects of BMS-986122 on 7-hydroxymitragynine between nanoBRET and TruPATH assay systems. Concentration-response curves were generated for 7-hydroxymitragynine in the presence of vehicle (● NanoBRET, ■ TruPATH), 1.0 μM (● NanoBRET, ■ TruPATH), and 10 μM (● NanoBRET, ■ TruPATH) BMS-986122 when MOR signaled through GαoA **(A)**, GαoB **(B)**, and Gαi3 **(C)**. **(D)** Comparison of the relative transduction coefficients of 7-hydroxymitragynine induced by 10 μM BMS-986122 when MOR signaled through GαoA, GαoB, and Gαi3 between nanoBRET and TruPATH assay systems. While assay sensitivity varied between nanoBRET and TruPATH for different Gα subunits, $\Delta\log(\tau)$ values calculated were not significantly different from one another (ns denotes $p > 0.05$, Student's t test). All plotted points are the mean values ± SEM from at least three independent experiments performed in duplicate.

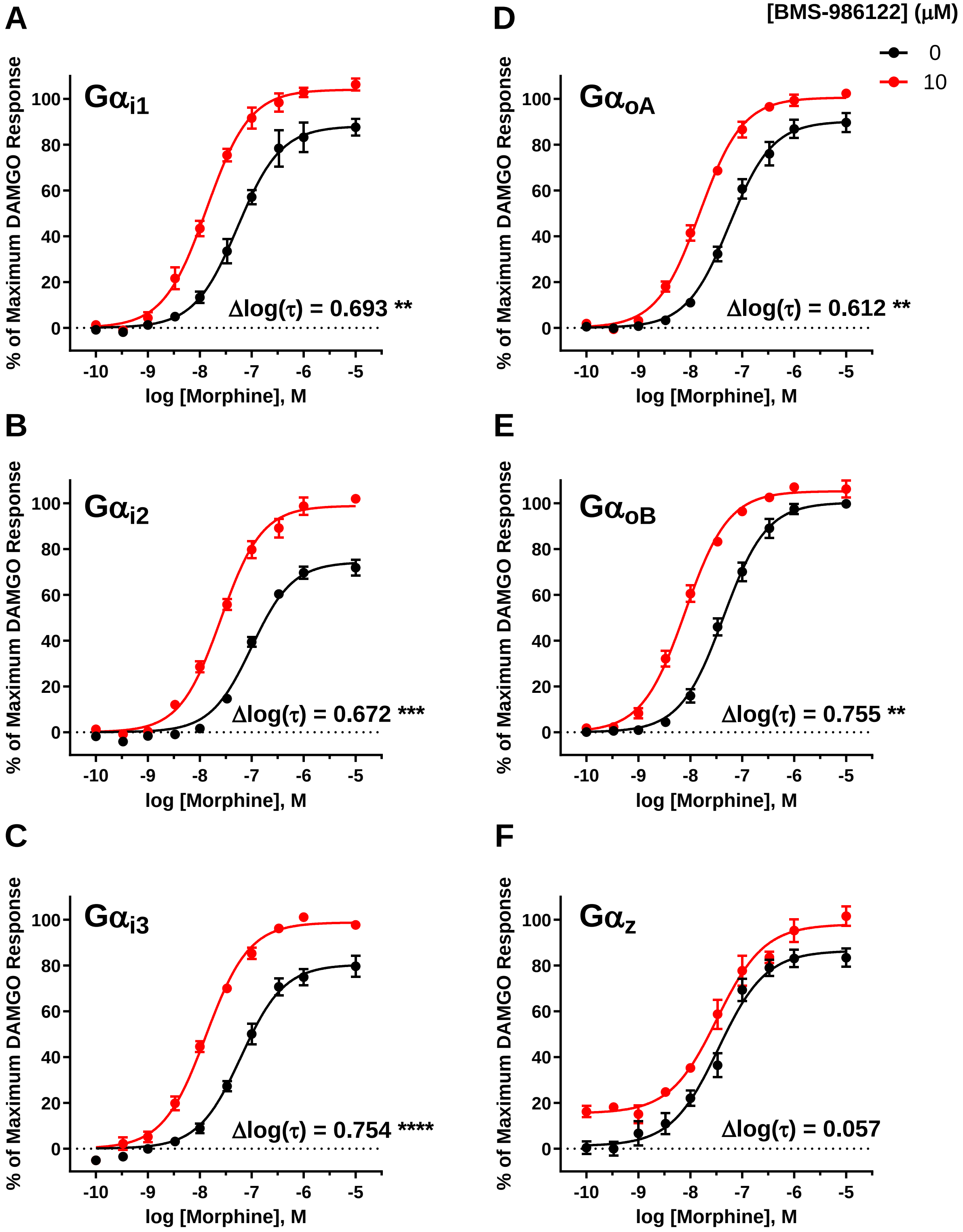

A
B
C
D
E
F
[BMS-986122] (μM)
0
10
Gαi1
Gαi2
Gαi3
GαoA
GαoB
Gαz
% of Maximum DAMGO Response
log [Morphine], M
Δlog(τ) = 0.693 **
Δlog(τ) = 0.672 ***
Δlog(τ) = 0.754 ****
Δlog(τ) = 0.612 **
Δlog(τ) = 0.755 **
Δlog(τ) = 0.057

Figure S4. Effect of 10 µM BMS-986122 on morphine when MOR signaled through different Gα subunits. Concentration-response curves were generated for morphine treated either with vehicle (●) or 10 uM BMS-986122 (●) when MOR signaled through Gαi1 **(A)**, Gαi2 **(B)**, Gαi3 **(C)**, GαoA **(D)**, GαoB **(E)**, and Gαz **(F)**. There were no statistically significant differences in relative transduction coefficients calculated for Gαi/o subunits, but all Gαi/o subunits were significantly different than Gαz (* denotes $p<0.05$, ** $p<0.01$, *** $p<0.001$, **** $p<0.0001$, one-way ANOVA with Tukey's post-hoc test). All points are the mean values ± SEM from at least three independent experiments performed in duplicate. $\Delta\log(\tau)$ values reported with standard errors can be found in Table 5.

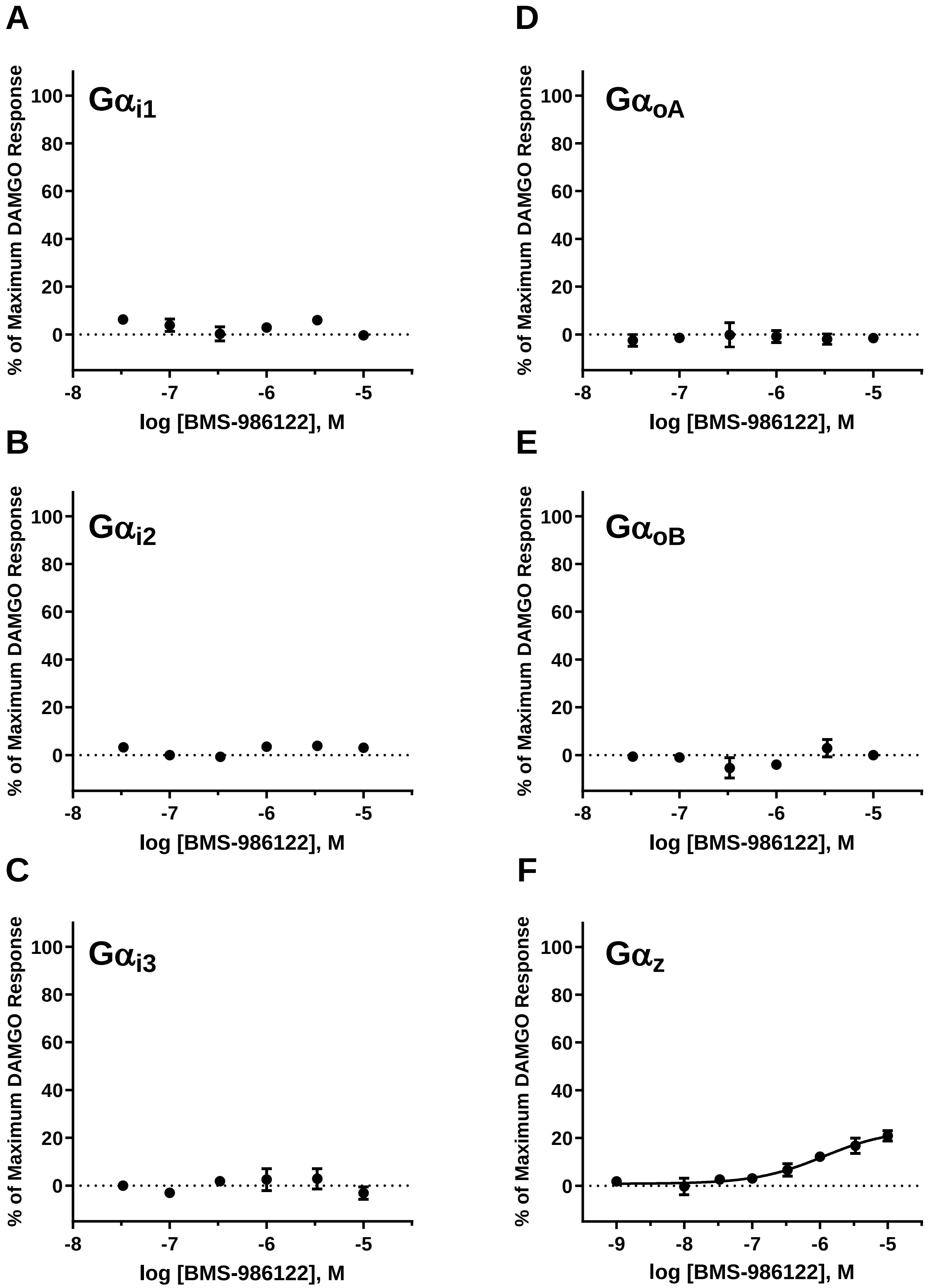
A
Gα$_{i1}$
% of Maximum DAMGO Response
100
80
60
40
20
0
-8
-7
-6
-5
log [BMS-986122], M
B
Gα$_{i2}$
% of Maximum DAMGO Response
log [BMS-986122], M
C
Gα$_{i3}$
% of Maximum DAMGO Response
log [BMS-986122], M
D
Gα$_{oA}$
% of Maximum DAMGO Response
log [BMS-986122], M
E
Gα$_{oB}$
% of Maximum DAMGO Response
log [BMS-986122], M
F
Gα$_{z}$
% of Maximum DAMGO Response
-9
-8
-7
-6
-5
log [BMS-986122], M

Figure S5. Titrations of BMS-986122 when MOR signaled through different Gα subunits.

Concentration-response data was collected for BMS-986122 when MOR signaled through Gαi1 **(A)**, Gαi2 **(B)**, Gαi3 **(C)**, GαoA **(D)**, GαoB **(E)** and Gαz **(F)**. All plotted points are the mean values ± SEM from at least three independent experiments performed in duplicate.
